# Simple Detection of Unstained Live Senescent Cells with Imaging Flow Cytometry

**DOI:** 10.3390/cells11162506

**Published:** 2022-08-12

**Authors:** Marco Malavolta, Robertina Giacconi, Francesco Piacenza, Sergio Strizzi, Maurizio Cardelli, Giorgia Bigossi, Serena Marcozzi, Luca Tiano, Fabio Marcheggiani, Giulia Matacchione, Angelica Giuliani, Fabiola Olivieri, Ilaria Crivellari, Antonio Paolo Beltrami, Alessandro Serra, Marco Demaria, Mauro Provinciali

**Affiliations:** 1 Advanced Technology Center for Aging Research, IRCCS INRCA, 60121 Ancona, Italy; 2 Department of Life and Environmental Sciences, Polytechnical University of Marche, 60121 Ancona, Italy; 3 Department of Clinical and Molecular Sciences, DISCLIMO, Polytechnical University of Marche, 60121 Ancona, Italy; 4 Center of Clinical Pathology and Innovative Therapy, IRCCS INRCA, 60121 Ancona, Italy; 5 Department of Medicine (DAME), University of Udine, 33100 Udine, Italy; 6 Luminex B.V., Het Zuiderkruis 1, 5215 MV ‘s-Hertogenbosch, The Netherlands; 7 European Research Institute for the Biology of Ageing (ERIBA), University Medical Center Groningen (UMCG), 9713 AV Groningen, The Netherlands

**Keywords:** cellular senescence, imaging flow cytometry, senolytics, replicative senescence, artificial intelligence and machine learning

## Abstract

Cellular senescence is a hallmark of aging and a promising target for therapeutic approaches. The identification of senescent cells requires multiple biomarkers and complex experimental procedures, resulting in increased variability and reduced sensitivity. Here, we propose a simple and broadly applicable imaging flow cytometry (IFC) method. This method is based on measuring autofluorescence and morphological parameters and on applying recent artificial intelligence (AI) and machine learning (ML) tools. We show that the results of this method are superior to those obtained measuring the classical senescence marker, senescence-associated beta-galactosidase (SA-β-Gal). We provide evidence that this method has the potential for diagnostic or prognostic applications as it was able to detect senescence in cardiac pericytes isolated from the hearts of patients affected by end-stage heart failure. We additionally demonstrate that it can be used to quantify senescence “in vivo” and can be used to evaluate the effects of senolytic compounds. We conclude that this method can be used as a simple and fast senescence assay independently of the origin of the cells and the procedure to induce senescence.

## 1. Introduction

Cellular senescence is a response to a variety of stresses and certain physiological processes characterized by a state of irreversible cell cycle arrest, induction of a secretome composed of bioactive molecules, macromolecular damage, and altered metabolism [1]. In culture, other features of senescent cells that have been well-known for a long time include an enlarged and flattened morphology [2] and increased levels of autofluorescence [3].

Senescent cells accumulate with aging in several tissues, contributing to a wide spectrum of aging phenotypes and diseases [4,5,6]. With the growing interest in therapeutic solutions that reduce the burden associated with accumulating senescent cells to improve health in aging and age-related diseases, the accurate detection of senescent cells is essential.

Given the heterogeneity of the cellular senescence phenotype, a universal single marker for the identification of senescent cells is lacking. For this reason, the recognition of senescent cells is based on multiple biomarkers, rendering it a time-consuming and labor-intensive task [7,8,9]. Increased activity of the senescence-associated beta-galactosidase (SA-β-Gal)—the first identified senescence marker [10]—by cytochemical or flow cytometry methods remains the most common readout for the detection of senescent cells [11]. Although high SA-β-Gal is measured in most senescent cells, it is not specific [9], and microscopy detection may suffer due to subjective interpretation of the images. The development of a flow cytometry method based on incubation with a fluorogenic substrate for SA-β-Gal, C_12_FDG, has provided a high-throughput alternative to the cytochemical method that reduces subjective biases [12]. This method can be combined with the measurement of altered morphology typical of senescent cells [13,14]. However, conventional flow cytometry cannot discriminate large senescent cells from multiplets of proliferating cells, thus leading to biases in the analysis of mixed populations. Moreover, this method requires complex and time-consuming experimental procedures, which hinders accuracy, reproducibility and its use for a large number of samples. 

Imaging flow cytometry (IFC) is a powerful tool that can produce snapshots of cells flowing in a fluid. This extra capability distinguishes it from conventional flow cytometry and allows us to combine features of both flow cytometry and fluorescence microscopy with advances in data-processing algorithms [15]. IFC has the unique capability of identifying collected events employing their optical images, a feature that is extremely useful in the study of cells displaying morphological changes. IFC has been previously adapted to detect SA-β-gal-stained cells [16], but the method was based on the same protocol of the cytochemical assay, and thus encounters similar problems as those listed above. More recently, a conventional flow cytometry approach has been proposed to detect senescent human mesenchymal stromal cells based on autofluorescence emission [17]. The autofluorescence signal is attributed to the presence of metabolic changes and lipopigments that accumulate in senescent cells [18,19]. However, this method does not account for the overlap of large senescent cells with multiplets, and it is unknown if autofluorescence can be used to identify other types of senescent cells in flow cytometry. 

Here, we propose a new, simple and broadly applicable IFC method based on combined measurement of autofluorescence and morphological parameters to quantify cellular senescence in living cellular populations. 

## 2. Results

### 2.1. Analysis of Senescent Cells Is Biased by Multiplets That Can Be Removed with a Specific Gating Strategy

**Figure 1:**
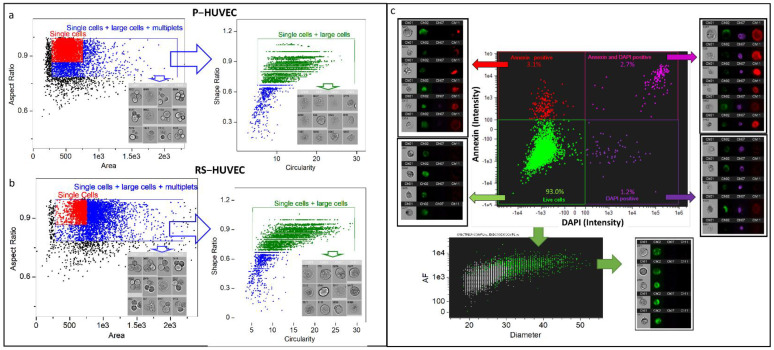
**Gating strategy used for the analysis of senescent cells.** Representative example from the HUVEC model of the gating strategy applied to proliferating (**a**) and senescent (**b**) cells. In the example, the conventional “Single cells” gate used in imaging flow cytometry to select proliferating cells (red color) is not appropriate when senescent cells are present in the sample, as the population is shifted to right in the dot plot “Aspect Ratio” vs. “Area”. However, extending this gate to the right leads to the inclusion of multiplets in the analysis (single cells + large cells + multiplets gate, blue color). A further gate (green gate) set in the high “Circularity” and high “Shape ratio” can quite completely clean the population of multiplets and other artifacts independently by the status of the cells. Cell images below each gate represent the largest events detected in the respective gate. Further selection of live cells (**c**) can be performed by staining the cells with Annexin-APC and DAPI to remove apoptotic and dead cells. The phenotype of the remaining cells can be monitored based on autofluorescence (intensity of channel 2, band 480–560 nm) and diameter ((width + height) /2)) parameters. In the bottom of panel **c**, there is an overlayed representative dot plot of a senescent (green events) and proliferating (gray events) sample showing that both autofluorescence and diameter are different between the two samples. P = proliferating; RS = replicative senescent; HUVEC= human umbilical vein endothelial cells; HMSC = human bone marrow mesenchymal stem cells.

Doublets and multiplets are a critical problem in the analysis of senescent cells by flow cytometry because the enlarged morphology of senescent cells shifts the population into the region occupied by cell aggregates. The exclusion of doublets and multiplets is usually performed in IFC by gating focused cells in the aspect ratio (AR) vs. area dot plot. In order to estimate the impact of the change in morphology to this conventional strategy, we collected representative AR vs. area dot plots obtained from different human and mouse cell models. As replicative senescence models, we used human umbilical vein endothelial cells (HUVEC) and bone marrow mesenchymal cells (MSC). As DNA damage induced senescence models, we used mouse ear fibroblasts (MearF) induced to senescence by continuous exposure (1 week) to 75 nM doxorubicin (DOX) or mitomycin C (MMC), as well as human dermal fibroblasts (HuDe) induced to senescence by continuous exposure to MMC (75 nM, 1 week). The senescent phenotype was confirmed in all models by the histochemical detection of SA-βgal activity and p16 and p21 mRNA expression (Supplementary Figure S1). When we attempted to gate senescent cells with the conventional “Single Cells” gate used for proliferating cells, we observed that most events were shifted to the right of the dot plot (Figure 1 and Figure S2). Thus, using the conventional gating strategy results in a loss of significant events (i.e., the largest cells) in the region of multiplets, which in turn are excluded to avoid false positives and biases. To avoid the loss of these events, we extended the gate to the multiplets region and applied a further gate in high “Circularity” and high “Shape ratio” (Figure 1c, right panels). As shown in Figure 1, this gate allows us to perform unbiased analysis without any loss of the events with the largest physical size and excluding multiplets. This is particularly important for unknown samples including both senescent and non-senescent cells. In a representative example with a merged file of proliferating and senescent HUVECs, we estimated that the loss of senescent large cells ranges from 10% to 43.2% with the conventional gating strategy and that the attempt to overcome this problem, slightly extending the gate to the right of the dot plot, results in the inclusion of an important amount of multiplets (above 2%) in the analysis (Supplementary Figure S3a). 

Since dead and apoptotic cells may also affect the morphological parameters of the population, we used combined staining with DAPI and annexin APC for their identification and removal (Figure 1c). To identify the parameters that could discriminate between senescent and proliferating cells in the resulting living population, we applied the Feature Finder Tool (IDEAS software) to merge files of the “live cells” population obtained from senescent and non-senescent samples. Following the “Feature Finder Wizard” instructions, we created a gallery of “true senescent” and “true non-senescent cells” by picking 20 cells in the extreme left and right of the “Area vs. AR” dot plots of the merged populations (Supplementary Figure S4). The Feature Finder ranks the top features based on Fisher’s discriminant ratio (Rd = Mean1 − Mean2/StdDev1 + StdDev2) (Supplementary Figure S4). Rd scores above 1.5 are widely accepted as a good indicator that the feature can discriminate the two groups tested. We found that two parameters can be used for this purpose in all models: (1) autofluorescence (AF), measured in the channel Ch02, band 480–560 nm; (2) width or height of the cells. These last two parameters were further combined in a single parameter named diameter ((D = width + height/2)). The resulting live cell population can thus be characterized by AF and D in all models, as shown by the overlayed dot plot of senescent and proliferating cells reported in Figure 1c.

### 2.2. Quantification of Cellular Senescence in Various Human and Murine Senescence Models

To improve the reproducibility of the present assay, we first quantified the increase in AF and D in senescent MearF, MSC, HuDe and HUVEC compared to the respective non-senescent population. 

**Figure 2:**
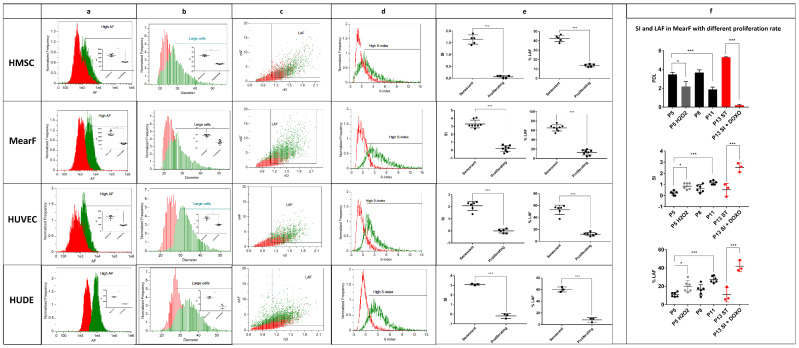
**Quantitative estimation of the senescent index (SI) and % of large autofluorescent cells (LAF) in various senescence models**. (**a**) Overlayed histograms and quantitative estimation of autofluorescence (AF) in proliferating (red) and senescent cells (green). All senescent samples display increased autofluorescence compared to non-proliferating samples. (**b**) Overlayed histograms and quantitative estimation of the diameter (D = (width + height)/2)) in proliferating (red) and senescent cells (green). All senescent samples display increased diameter compared to non-proliferating samples. (**c**) Overlayed representative dot plots of normalized autofluorescence (nAF = “AF”/“mean AF of proliferating samples”) vs. normalized diameter (nD = “D”/“mean D of proliferating samples”) showing that LAF increases in senescent samples (green events) compared to non-senescent samples (red events). The threshold of nAF was set at 1.5 whereas the threshold of D was set at 1.1 for all samples. (**d**) Overlayed representative histograms of the SI (SI = ((nAF − 1) + 5 × (nD − 1))/2) in proliferating (red) and senescent cells (green). (**e**) Quantitative estimation of SI and LAF in proliferating and senescent HMSC (*n* = 5), MearF (*n* = 8), HUVEC (*n* = 6) and HuDe (*n* = 3). (**f**) Comparison of SI and LAF in MearF with different population doubling level (PDL). SI and LAF significantly increase after stressing conditions that decrease PDL, such as treatment with H_2_O_2_ (250µM H_2_O_2_ × 2 h + 1 week of resting, *n* = 6) or at late passages (P11, *n* = 6) compared to early passages (P5, *n* = 6). Spontaneous transformation of one of the cultures (P13 ST, *n* = 3 replicates from the same culture) resulted in a strong increase in PDL and a parallel decrease in SI and LAF. Treatment of P13 ST with doxorubicin 75 nM × 1 week (P13 ST + DOXO, *n* = 3 replicates from the same culture) strongly increased both SI and LAF. MearF = mouse ear fibroblasts; HUVEC = human umbilical vein endothelial cells; HMSC = human bone marrow mesenchymal stem cells; HuDe = human dermal fibroblasts; * *p* < 0.05; *** *p* < 0.001 by Student’s *t* test.

The AF measured in the Ch02 channel increased approximatively 2-fold in replicative senescent MSC (mean ± SD = 1806 ± 143 vs. 992 ± 28, *p* < 0.001) and HUVEC (1915 ± 148 vs. 907 ± 69, *p* < 0.001), and 3-fold in DOX-induced senescent MearF (3789 ± 505 vs. 1309 ± 157, *p* < 0.001) and MMC-induced HuDe (8770 ± 435 vs. 2892 ± 100, *p* < 0.001) compared to the respective non-senescent populations (Figure 2a). Similarly, the D of the cells increased approximatively 1.2-fold in replicative senescent HUVEC (mean ± SD = 32.2 µm ± 1.1 vs. 26.7 µm ± 0.5, *p* < 0.001), DOX-induced senescent MearF (29.5 µm ± 0.9 vs. 24.4 µm ± 1.3, *p* < 0.001) and MMC-induced senescent HuDe (34.4 µm ± 0.6 vs. 26.7 µm ± 1.6, *p* < 0.01), and 1.1-fold in MSC (28.6 µm ± 0.32 vs. 25.0 µm ± 0.15, *p* < 0.001) compared to the respective non-senescent populations (Figure 2b).

In the case of MearF, the AF of samples treated with DOX was corrected for potential interference due to DOX-specific fluorescence [20], which displayed the maximum signal in Ch04 (Supplementary Figure S5). Since we treated it with an extremely low concentration of DOX (75 nM) and removed the DOX-containing medium at least 2–6 days before the acquisitions, the remaining signal due to the DOX entrapped within the cells should be minimal or negligible. To prevent potential bias, we also made a precautionary correction by compensation using samples treated with a massive DOX concentration (100 µM). The potential spillover from Ch04 to Ch02 was thus calculated and subtracted to the AF (Ch02). The increased AF in DNA-damage-induced senescent MearF was additionally confirmed by treatment with MMC (75 nM for 1 week). Indeed, MMC does not display intrinsic AF visible in the Ch02 channel (Supplementary Figure S6) and, similarly to DOX, is known to induce senescence by DNA damage [21]. As expected, samples treated with MMC showed a mean increase in AF intensity above 3000 (Supplementary Figure S6), similar to the results observed with DOX (Figure 2a), thus confirming that the increased AF of senescent MearF is due to processes associated with senescence rather than to the intrinsic properties of the drug. 

We further computed a normalized AF (nAF = “AF”/“mean AF of proliferating samples”) and normalized diameter (nD = “D”/“mean D of proliferating samples”) in all samples. Independently from the cell type or the senescence inducer, we observed that samples from proliferating cells displayed relatively few events above 1.5 nAF and 1.1 nD compared to senescent cells. Based on this observation, we established a gate (Figure 2c) with the thresholds of 1.5 nAF and 1.1 nD for all models and named the cells selected by this gate as large autofluorescent senescent cells (LAFs). To obtain a sensitive estimation of senescence valid for all models, we also computed an index, named the S-index (SI = ((nAF − 1) + 5 × (nD − 1))/2) (Figure 2d). The SI uses a weight factor = 5 to compensate the general fivefold higher increase in nAF compared to the one of nD in senescent cells. Both SI and % of LAFs were abundantly and significantly higher in senescent cells (*p* < 0.001 in all models for both parameters) compared to non-senescent cells (Figure 2e, Supplementary Figure S6d). Importantly, the SI of non-senescent cells remains stable around 0 independently from the model (mean ± SD = 0.2 ± 0.4, 0.0 ± 0.2 and 0.1 ± 0.1, −0.2 ± 0.2 in MearF, HUVEC, MSC and HuDe, respectively), whereas the SI of senescent cells increases at least above 1.3 (mean ± SD = 3.3 ± 0.5, 2.1 ± 0.4, 1.6 ± 0.2, 3.0 ± 0.1 in MearF, HUVEC, MSC and HuDe, respectively). The % of LAF (mean ± SD) was also very similar and estimated at around 10% in proliferating cells (mean ± SD = 13.0 ± 5.6, 12.12 ± 2.8, 13.8 ± 1.5, 8.15 ± 3.7 in MearF, HUVEC, MSC and HuDe, respectively) and at least above 35% in senescent cells (mean ± SD = 65.4 ± 6.7, 52.8 ± 7.9, 42.4 ± 3.5, 56.2 ± 4.2 in MearF, HUVEC, MSC and HuDe, respectively). 

In order to further exclude the potential contribution of DNA-inducing reagents to autofluorescence, we also measured SI and LAF in MearF at 3 and 8 days post exposure to ionizing radiation (10 Gy). We observed a gradual and significant increase in both SI and LAF in these samples compared to untreated proliferating cells that was further confirmed by Spider-βGAL staining at 8 days (Supplementary Figure S6).

We also measured SI and LAF in MearF treated with stressing conditions that are known to induce a decrease in the proliferation rate and to partially increase the number of senescent cells (Figure 2f), such as treatment with H_2_O_2_ and serial replicative stress. We found a significant decrease in PDL and a parallel significant increase in SI and % LAF in MearF after treatment with H_2_O_2_ (250 µM H_2_O_2_ for 2 h followed by 1 week in normal medium) as well as after continuous serial passages (P11) vs. cells at early passage (P5). Importantly, SI and % of LAF were reversed to levels similar to P5 in one of our MearF cultures that underwent spontaneous transformation and immortalization at passage 13 (P13 ST). Moreover, further treatment of this culture with DOX induced a strong increase in both SI and % LAF (Figure 2f).

### 2.3. Quantification of Cellular Senescence in Ex-Vivo Samples

**Figure 3:**
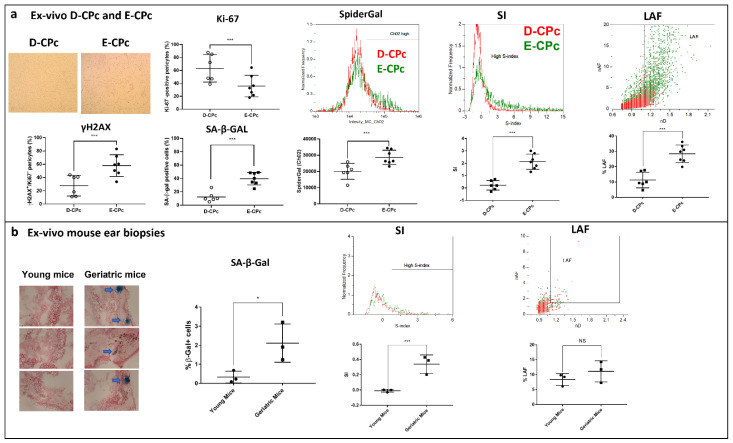
**Estimation of senescence by imaging flow cytometry in “ex vivo” human and mouse samples** (**a**) Estimation of senescence by imaging flow cytometry (IFC) in ex vivo cardiac pericytes (CPcs). On the left of the panel, representative microscope images are shown, and senescence (SA-β-gal and γH2AX positive cells) and proliferative (Ki-67 positive cells) markers showing that CPcs from patients undergoing cardiac transplantation (E-CPc, *n* = 7) display a high degree of senescence compared to CPcs from healthy donors (D-CPcs, *n* = 6). A representative histogram and the quantification of senescence performed by flow cytometry Spider-βGal assay (SpiderGal, middle of the panel) also confirmed the higher degree of senescence of E-CPcs. In agreement with these biomarkers, we detected a significant increase in LAF and SI in E-CPcs compared to D-CPcs by IFC (right of the panel). (**b**) Estimation of senescence by IFC in cells isolated from mouse ear biopsies. Ear biopsies taken from geriatric mice display and increased staining for SA-β-gal (left of the panel). SI also significantly increased in cells isolated form ear biopsies taken from geriatric mice vs. those from young mice. This difference was not detected for LAF. * *p* < 0.05; *** *p* < 0.001 by student’s *t* test.

To test our method in a clinically relevant model, we collected samples of cardiac pericytes (CPcs) obtained from patients undergoing cardiac transplantation (E-CPcs) or from healthy donors (D-CPcs). Previous investigation in this model provided preliminary evidence that E-CPcs residing in ischemic failing human hearts are enriched by senescent cells [22,23]. E-CPcs were characterized by reduced proliferation rate (significantly reduced % of Ki-67 positive cells) and increased % of phosphorylated histone H2AX (γH2AX), as well as by increased SA-β-gal-positive cells (measured by microscopy) and activity (measured by Spider-βGAL flow cytometry assay), reminiscent of senescence (Figure 3a). As expected, SI and % LAF of E-CPcs were significantly higher compared to D-EPcs (*p* < 0.001) with a mean SI (± SD) of 0.2 ± 0.4 and 2.2 ± 0.6 as well as a mean % LAF of 11.3 ± 5.1 and 28.4 ± 5.7 in D-CPcs and E-CPcs, respectively (Figure 3a). Importantly, staining of these cells with Spider-βGAL provided an additional proof of principle that also in these samples, the cells in the “High Area region” are mostly represented by senescent cells (Supplementary Figure S3).

To test our method with samples reflecting an “in vivo” condition, we analyzed cells immediately isolated from ear biopsies (2 mm) of young and geriatric mice. In order to verify that the ear biopsies taken from geriatric mice display an accumulation of senescent cells, we carried out SA-β-gal histochemistry assay in one biopsy. The SA-β-gal assay showed increased staining in the tissues of geriatric mice compared to those of young mice, possibly reflecting an accumulation of senescent cells (Figure 3b). Another biopsy, taken from the same ear, was used to rapidly isolate live cells and to run our IFC method in this fresh material. Although the number of cells recovered from the tissue was extremely low (100–200 cells per samples), it was possible to perform the measurement of both SI and LAF. We found that the SI significantly increased in the cells isolated from the biopsies of geriatric mice with a mean SI (±SD) of 0.33 ± 0.12 compared to the −0.03 ± 0.02 measured in the cells from the young mice biopsies (Figure 3b). Conversely, we failed to detect significant differences in the % LAF between the two groups (mean % LAF ± SD was 6.11 ± 2.0 in cells from young and 7.31 ± 11.14 in those from geriatric biopsies) (Figure 3b).

### 2.4. Comparison of the Effect of Common Senolytics on Mouse Senescent Fibroblasts

Screening of candidate senolytic drugs are usually based only on the assessment of selective death in senescent cells. However, information on potential induction of apoptosis and on the phenotype of cells surviving the treatment are collected later with additional assays. We supposed that our IFC method could be useful to validate candidate senolytics, to determine if cell death occurs by apoptosis, as well as to compare the phenotype (e.g., morphology) of the cells remaining alive after treatment.

**Figure 4:**
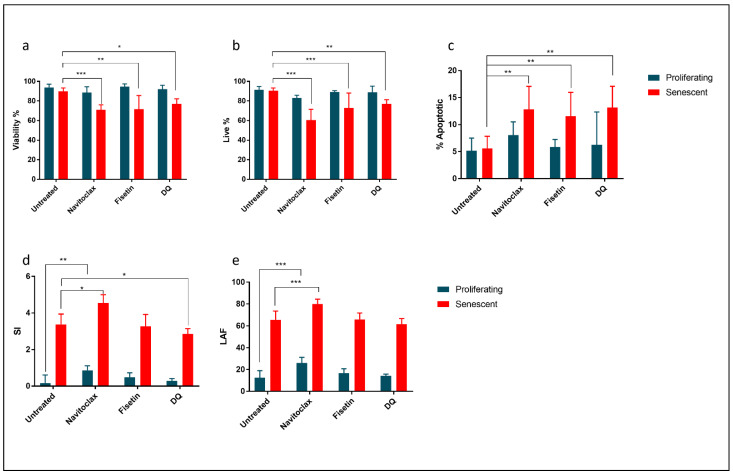
**Treatment of proliferating or senescent mouse ear fibroblasts (MearF) with various senolytics**. (**a**) Viability assay performed by Trypan Blue exclusion showing that exposure (48 h) to navitoclax (10 μM), fisetin (10 μM) or the combination of dasatinib + quercetin (DQ) (100 nM + 10 μM) reduces the viability only in senescent fibroblasts. (**b**) Live cells estimated by imaging flow cytometry (% of cells negative to DAPI and annexin in the IFC assay) also significantly decrease only in senescent fibroblasts. (**c**) Apoptotic cells (% of cells positive to annexin and negative to DAPI) significantly increased after treatment with all senolytics. (**d**) The senescence index (SI) estimated by IFC significantly decreases in senescent samples after treatment with DQ. Conversely, SI increases after treatment with navitoclax. (**e**) The % of large autofluorescent cells (LAFs) significantly increase in senescent and proliferating samples only after treatment with navitoclax2.5. Quantification of senescent cells estimated by artificial intelligence (AI) and machine learning (ML) * *p* < 0.05; ** *p* < 0.01; *** *p* < 0.001 by ANOVA followed by post hoc.

We tested our method in samples of non-senescent and DOX-induced senescent MearF treated with some common senolytics (Navitoclax, Fisetin and DQ). All these compounds were able to significantly decrease the viability of senescent cells (Figure 4a) by trypan blue assay. In agreement with the results of the trypan blue assay, we observed a significant decrease in the % of live cells (% of cells negative to DAPI and annexin) (Figure 4b) and a significant increase in apoptosis (% of cells positive to annexin and negative to DAPI) (Figure 4c) only in senescent cells.

Unexpectedly, the measurement of SI and LAF (restricted to the live cell population) provided evidence of differential effects of senolytics. Indeed, only DQ induced a small but significant decrease in SI in senescent cells, whereas Navitoclax induced a paradoxical increase in SI both in senescent and proliferating cells (Figure 4d). Navitoclax also induced a small but significant increase in LAF (Figure 4e) in both senescent and non-senescent cells, while Fisetin treatment did not affect either SI or LAF. 

**Figure 5:**
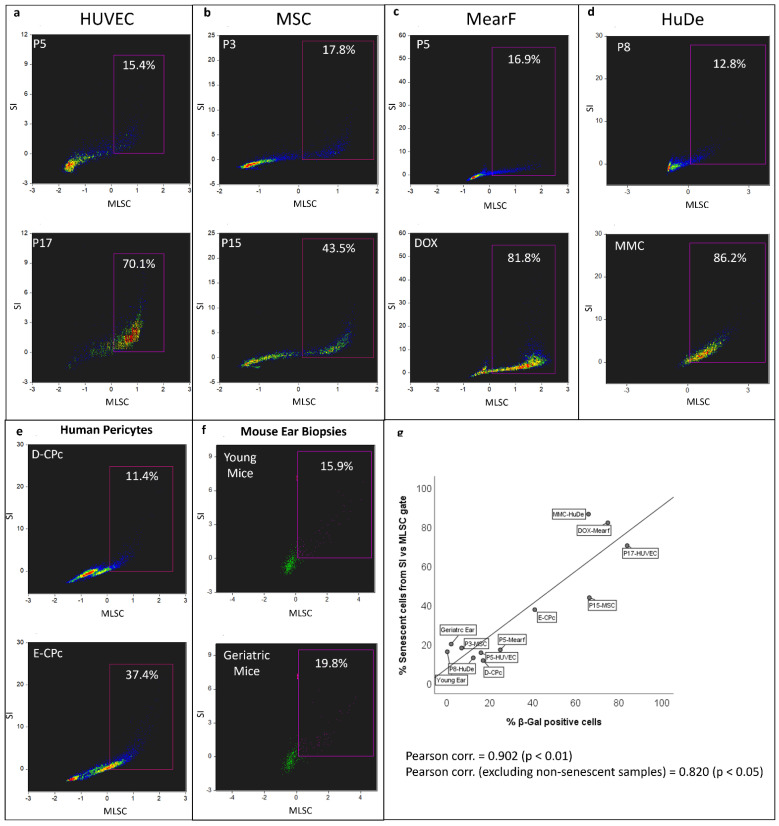
**Estimation of senescence by imaging flow cytometry implemented with artificial intelligence and machine learning in different cellular models**. Scatter plots of the super-feature derived from AI and ML elaboration, “ML Senescence Classifier” (MLSC), versus Senescence Index (SI). Panel (**a**), human umbilical vein endothelial cells (HUVEC) at passage P5 and passage 17 (P17); panel (**b**), human mesenchymal stem cells (MSC) at passage (P5) and passage (P15); panel (**c**), murine ear fibroblasts (MearF) at passage 5 (P5) and after treatment with Doxorubin (DOX); panel (**d**), human dermal fibroblasts (HuDe) at passage 8 (P8) and after treatment with Mitomycin C (MMC), panel (**e**), “ex-vivo” cardiac pericytes obtained from one healthy donor (D-CPc) and from one patient undergoing cardiac transplantation (E-CPc); (**f**) “ex-vivo” cells immediately extracted from ear biopsies of young and geriatric mice; panel (**g**), correlation between the senescent cells gated from the SI vs. MLSC scatter plot versus the % of β-galactosidase (β-Gal) positive cells assessed by light microscopy. Pearson and Spearman correlations (both *p* < 0.01) are shown in the graph.

We explored whether the newly implemented tools of artificial intelligence (AI) and machine learning (ML) could be used to identify unstained living senescent cells. The datafiles were analyzed with the IDEAS software to calculate the SI and the results were used as a basis to export the “Live cell” population after excluding Annexin and DAPI positive cells (see Figure 1c). A schematic representation of the workflow followed to analyze IFC data integrating AI and ML is described in Supplementary Figure S7. The datafiles were subject to a first classifier generated in Amnis AI able to generically distinguish objects with an integral image from objects whose image is clipped. The identification of a clipped object was applied regardless of its image consisting of either one or multiple cells or debris (Supplementary Figure S8A). The objects classified as non-clipped were thus exported as a separate datafile and subsequently analyzed with a second classifier able to separate single cells from aggregates of different sorts (Supplementary Figure S8B). To quantify the difference between senescent and non-senescent cells, a super-feature was calculated using the ML module of IDEAS 6.3. A new datafile for each experimental model of senescence (MearF, HUVEC, MSC, Cardiac pericytes) was created by merging Amnis AI-classified single non-senescent cells with their senescent counterparts. The resulting file was initially analyzed using the template used to extrapolate the SI. Two truth populations were defined in order to identify senescent (“S TRUTH”) versus non-senescent (“NON S TRUTH”) cells. The “NON S TRUTH” consisted of cells from the non-senescent control characterized by an SI ≤ 0.8. The “S TRUTH” consisted of cells from cultures of induced senescence with an SI ≥ 1.2 (Supplementary Figure S9, Panel A). Based on these criteria, the ML algorithm calculated the super-feature that maximally separates each truth population from the other. This classifier is based on user-defined and/or ML-generated single features, which are ranked and combined by a Linear Discriminant Analysis. The calculated classifier, called “ML Senescence Classifier” (MLSC), is >0 for senescent cells and <0 for non-senescent cells (Supplementary Figure S9, Panel B). This parameter was then used to represent the experimental cell samples on scatter plots against the SI (Figure 5). In these graphs, senescent cells have been defined as expressing values of MLSC and SI simultaneously higher than zero, whereas non-senescent are described by both parameters lower than zero.

The AI and ML tool applied to representative samples from each model shows the effectiveness of the proposed technique in discriminating senescent cells both in replicative, damage-induced and “ex-vivo” model of senescence (Figure 5). Moreover, the % of cells gated in the senescent zone was highly correlated with the respective SA-β-gal staining of the representative samples, even excluding non-senescent controls (Figure 5). Based on the extreme events from the population density observed in the SI vs. MLSC scatter plots, we also calculated a second classifier (MLSC2) and plotted SI vs. MLSCs. With the noted exception of the samples from MearF, these new scatter plots provided a better separation between senescent and non-senescent cells and uncovered the presence of various sub-populations, which may be related to the progressive development of the senescent phenotype (Supplementary Figure S10). These scatterplots of SI vs. MLSC2 seem particularly efficient to separate senescent cells in the HUVEC model and to identify several sub-populations in the MSC model as well as in “ex-vivo samples” from human pericytes (Supplementary Figure S10).

## 3. Discussion

We here provide a fast and simple method to identify live unstained senescent cells by imaging flow cytometry. The method is based on the normalized autofluorescence (nAF) and diameter (nD), which can be easily computed once AF and D are calculated in samples of normal proliferating cells. The use of nAF and nD instead of their absolute values is useful for comparative purposes when cells of different origin (tissues and species) treated with different stimuli (DNA damaging agents, H_2_O_2_ or replicative stress) are used. The two parameters (SI and % LAF) that we used to quantify senescent cells are also simple in their interpretation as SI ≈ 0 in non-senescent cells and increases to 1.5–4 in senescent cells. Similarly, % LAF is ≈ 5–15% in non-senescent cell cultures and increases to 40–80% in cultures of senescent cells. Importantly, the thresholds used to gate LAF have been kept the same for all models tested in this work (nAF = 1.5 and nD = 1.1). Increasing the thresholds of nAF and nD above these levels increases the specificity to discriminate senescent from non-senescent samples (data not shown) but decreases the sensitivity, thus increasing the rate of false negative results, which may be critical in the case of limited amounts of sample. We deem that the reported thresholds may offer an optimal compromise, but the method can be further optimized on the basis of specific requirements. Our results extend to various models of senescence and, to cells of different origin, the previous observation made with conventional flow cytometry in human mesenchymal stromal cells [17]. Compared to conventional flow cytometry, our approach has the advantage of accurately discriminating and removing multiplets from the analysis without loss of events of large physical size. Indeed, the enlarged morphology of senescent cells “in vitro” is a distinctive feature and it has been widely documented [24,25]. By taking advantage of the fact that the cells assume a circular shape once suspended, we were able to obtain a quantitative estimate of the diameter of the cells in different models (HUVEC, MearF, HuDe and MSC). The relative increase in nD in senescent cells was found to be around 1.1–1.2-fold, but some cells showed even a 2-fold increase compared to proliferating cells. Thus, the combination of this morphological change with AF is very powerful for the identification of senescent cells. AF was used several years ago to discriminate and sort human diploid fibroblasts in late-passage cultures [26], but only recently this feature has begun to be appreciated for the identification of mesenchymal senescent cells by microscopy or flow cytometry [17,18,19]. The increased AF of senescent cells has been attributed to an increase in the fluorescent cellular organelles (i.e., mitochondria and lysosomes), flavin adenine dinucleotide (FAD) as well as to the accumulation of lipopigments or lipofuscins [18,27,28,29]. We chose the channel band (Ch02, 480–560 nm) for the collection of AF signals on the basis of the maximum signal observed in our preliminary experiments. The contribution to the AF signals collected from other channels (Ch03 and Ch04) was indeed very low and displayed a large variation between cultures (data not shown), as also confirmed by others [17]. 

Using the settings described above, we were able to rapidly discriminate senescent vs. non-senescent MearF, HUVEC, HuDe and MSC. Importantly, we also applied this method to “ex-vivo” samples of CPc from donors and explanted hearts. It has been previously shown that E-CPc residing in ischemic failing human hearts are senescent and display altered mechano-transduction properties [22,23]. Following a few days in culture, we observed that these cells are similar to senescent cells with the typical morphological changes and an increase in many markers associated with cellular senescence. Our method was able to differentiate with great sensitivity the D-CPcs from the E-CPcs based on LAF and SI, which in turn were significantly higher in E-CPc as expected. The flow cytometry SpiderGal assay provided similar results but with a lower fold change in the signal, suggesting an overall lower sensitivity compared to our method. Although we disposed only of a limited sample of patients, the difference was large enough to suggest that the sample size requirement for this characterization is relatively low and that these features may be distinctive of E-CPcs.

A slightly different picture emerged from the analysis of mouse cells immediately extracted from ear biopsies. Indeed, we found a higher SI in the cells isolated from the biopsies of geriatric mice compared to those isolated from young mice, but we did not detect any significant difference in LAF. Thus, AF was the major discriminating factor for the observed difference. This is not completely surprising as there is limited evidence that senescent cells can present an increased size “in vivo”, probably due to constraints related to the tissue architecture [8]. It is also expected that the percentage of senescent cells “in vivo”, even from old animals, is relatively low [16]. The results provide a preliminary proof of concept that the method may even be optimized for applications “in vivo”. Moreover, it should be relatively simple to improve the phenotyping with specific antibodies conjugated to fluorochromes that do not overlap with Ch02. A promising approach could be to use antibodies against dipeptidyl peptidase 4 (DPP4), which was recently found to be selectively expressed on the surface of senescent cells [30]. Emerging microfluidic tissue dissociation approaches may eventually be adapted to prepare isolated cells for IFC from small biopsies, thus providing a potential useful clinical tool for trials with senolytics or other treatments that are thought to affect the number of senescent cells in specific tissues. 

Importantly, the present method appears to also be sensitive to a partial induction of senescence. After 48 h of exposure to concentrations of H_2_O_2_ above 150 µM, mouse fibroblasts exhibit significant increases in SA-β-gal and a significant decline in the proliferation rate [31]. Since parts of the cells still retain the ability to proliferate, the decline in the proliferation rate is consistent with a partial and not complete induction of senescence. After replicating these experimental conditions (with 200 µM H_2_O_2_), we found that our method was able to sense this partial induction of senescence, as documented by the significant increase in SI and LAF in the H_2_O_2_-treated MearF compared to the respective untreated controls. Cells treated with ionizing radiation are reported to progressively develop the senescent phenotypes in the days post-irradiation [32]. Additionally, in this model, we were able to sensitively detect the development of senescence by measuring the SI and LAF of irradiated (10 Gy) MearF at 3 and 8 days post-irradiation (Supplementary Figure S6, panel g).

We also observed a significant increase in SI and LAF when the proliferation rate was decreased at advanced passages (P11). Primary mouse fibroblasts normally have a limited growth capacity but rare cases of spontaneous immortalization on prolonged passaging have been already reported [33,34]. Interestingly, when one of our cultures displayed a spontaneous transformation and immortalization, with a huge increase in the proliferation rate, we also observed a reversal of SI and LAF to the levels observed at P5. These results suggest that the method can sense even a relatively small change in the number of senescent cells in the population. 

In Table 1, we have resumed the capacity of the present method to discriminate between senescent and non-senescent cells in different models. From this table, it may appear that the methods perform better in DNA-damage-induced models than “ex-vivo” and “in vivo” models. However, it should be considered that the expected number of senescent cells is usually lower in “ex-vivo” and “in vivo” models. A similar consideration can be made for replicative models, as late passages in culture may still comprise a certain amount of non-senescent cells.

The major advantages of the present method compared with those based on conventional flow cytometry [35] is that we can accurately analyze the whole population of senescent cells excluding multiplets or other potential artifacts that cannot be discriminated without the imaging tool of IFC. Another approach, based on fluorescent ubiquitination-based cell cycle indicator (FUCCI) technology, was also developed to isolate live premature senescent cells induced by DOX treatment [36]. However, this method is based on the viral infection efficiency of the cells, while the method presented in this manuscript do not require any manipulation. Moreover, our approach requires much less time and operational procedures compared others based on iodixanol (OptiPrep) density gradient separation [37] or staining with C12FDG [38]. Conversely, the major disadvantage of our method compared to those cited above is that the IFC technology is currently limited to the analysis and does not allow us to isolate the gated cells population.

The possibility to quantify apoptotic and necrotic cells (using Annexin and DAPI staining) with this method is also useful in preliminary tests of putative senolytics “in vitro”. When we tested three common senolytics, Navitoclax [39], DQ [40] and Fisetin [41], we confirmed that all the compounds were able to reduce the % of live cells and increase the % of apoptotic cells in senescent MearF. In contrast with our expectations, none of the senolytics induced a decrease in LAF, and only DQ induced a decrease in SI in senescent cells. In the case of navitoclax, we also observed a paradoxical increase in LAF and SI both in senescent and non-senescent samples. This may appear paradoxical but not completely surprising, as the toxic effects of navitoclax are selective but not specific for senescent cells. This means that navitoclax, and likely other senolytics, may eventually induce a certain degree of stress even in proliferating cells. We observed a similar phenomenon in the past, treating endothelial cells with cytotoxic doses of zinc “in vitro” [14]. In agreement with this hypothesis, it was reported that esophageal cancer cells treated with Navitoclax displayed a senescent-like phenotype with arrested cell cycle, increased expression of p21 and decreased expression of phospho-Rb [42]. Despite a clear effectiveness in several pre-clinical studies [43], it is known that Navitoclax senolytic activity can be highly variable [44]. Considering our results and past observations regarding Navitoclax toxicity [45,46], its use “in vivo” should take into account potential side effects. A recent report also shows that Navitoclax at senolytic doses (short-term treatment) in old mice causes trabecular bone loss and impaired osteoprogenitor function [31]. Notably, we used relatively high concentrations of Navitoclax (10 µM) as the senolytic effect should be more pronounced at this concentration [47]. The use of lower concentrations might have reduced cellular stress and facilitated discerning the impact on survival and morphology.

We additionally explored the advantages offered by AI and ML in automated image analysis to quantify senescence in some representative samples from our models. To the best of our knowledge, this is the first attempt to combine AI, ML and IFC in the quantification of senescence in cell cultures. We here provide the first evidence that this technology can be used to discriminate senescent vs. non-senescent cells, especially when plotted against the SI (Figure 5). This may provide a useful tool for an absolute count of senescent cells in cell culture or unknown “ex-vivo” samples independently from their origin or nature and without need for any staining (excluding those used to exclude dead and apoptotic cells). The ML tool can additionally be optimized to identify subpopulations in senescent samples (Supplementary Figure S10). This is in agreement with the heterogeneity of senescence previously observed [48], but we will not be able to provide additional information on these subpopulations until a sorter is coupled to IFC. Anyway, the results confirm that AI and ML can be successfully applied in the quantification of senescence in unstained cell samples and also provide evidence of the heterogeneity of the cell cultures, especially when they acquire a senescent phenotype. 

The limitation of this methods is that the cells need to be detached and resuspended before the acquisition and that the method is destructive (cells cannot be recovered unless there is future implementation of sorting). Unfortunately, the present method cannot be combined with intracellular staining of conventional senescence markers (such as p16 and p21) because the protocol would require additional steps for fixation and permeabilization that are likely to affect the morphology [49] and the autofluorescent features [50] of the cells.

However, since the method does not use any stain (excluding DAPI and Annexin APC to select live cells), there is space for at least an additional fluorochrome, for example one conjugated to some recent identified membrane biomarkers of senescent cells [51]. This should improve the characterization of senescent cells and the identification of subpopulations in heterogeneous cell samples. Indeed, the observed presence of very large cells in senescent samples would deserve further investigation to understand if these cells could be more harmful than others, i.e., by assessing their production of secretory factors and resistance to apoptosis. The future implementation of our strategy in intelligent image-activated cell sorting [52] will likely allow us to isolate the largest cells or specific subpopulations of senescent cells for RNAseq or proteomics with the aim to develop biomarkers or targeted therapies.

In conclusion, this method allows a simple and fast senescence assay independent of the origin of the cells and the procedure to induce senescence. It can be used to check senescence in cell cultures or after induction as well as in cells “ex vivo” from patients or animals. 

## 4. Methods

### 4.1. Cell Culture and Growth Conditions

#### 4.1.1. Human Bone Marrow (BM)-Derived Mesenchymal Stem Cells (MSCs) and Human Umbilical Vein Endothelial Cells (HUVEC)

Human BM-derived MSCs were purchased from PromoCell (C-12974, donor 439z037.1, female, 30 years; PromoCell, Heidelberg, Germany) and maintained in Mesenchymal Stem Cell Growth Medium 2 (C-28009, PromoCell) at 37 °C in a humidified atmosphere containing 5% CO_2_. Cells were seeded at a density of 5000/cm^2^, and the medium was changed at 48 h intervals. When cells reached about 80% confluence, they were washed with PBS, detached with 0.25% trypsin -EDTA (ECB3052; EuroClone, Milano, Italy), and passaged. Cells were cultured until they arrested their replication (approximatively around the 15th passage), and replicative senescence was assessed by senescence markers. 

HUVECs, primary human umbilical vein endothelial cells, obtained from a pool of donors, were purchased from Clonetics (Lonza, Stein, Switzerland) and cultured in endothelial basal medium (EBM-2, CC-3156, Lonza) supplemented with SingleQuot Bullet Kit (CC-4176, Lonza) containing 0.1% human recombinant epidermal growth factor (rh-EGF), 0.04% hydrocortisone, 0.1% vascular endothelial growth factor (VEGF), 0.4% human recombinant fibroblast growth factor (rh-FGF-B), 0.1% insulin-like growth factor-1 with the substitution of arginine for glutamic acid at position 3 (R3-IGF-1), 0.1% ascorbic acid, 0.1% heparin, 0.1% gentamicin and amphotericin-B (GA-1000), and 2% fetal bovine serum (FBS). The cells were seeded at a density of 5000/cm^2^ in T75 flasks (Corning Costar, Sigma Aldrich, St. Louis, MO, USA). Replicative senescence (confirmed by senescence markers and proliferation arrest) was achieved after a number of replicative passages (approximatively around the 17th passage). 

#### 4.1.2. Human Dermal Fibroblasts (HuDe)

Primary human dermal fibroblasts (HuDe) were purchased from the Istituto Zooprofilattico Sperimentale (Brescia, Italy) as a pooled sample from female subjects (40 years).

Cells were cultured in minimum essential medium with Earl Salts (ECB2071L, Euroclone), with 10% fetal bovine serum, 1% penicillin (10,000 U/mL), 1% streptomycin (10 mg/mL) and 1% Amphotericin B (250 ug/mL) maintained in standard culture conditions at 37 °C in CO_2_ under a humidified atmosphere.

The cells were plated at a density of 11,000/cm^2^, the medium was replaced every two days, and cultures were passaged when cell density reached 80% confluence. Experiments were conducted with 8P fibroblasts.

Stress-induced premature senescence (SISP) was induced using DNA damaging agent mitomycin (MMC) 75 nM for one week. Subsequently, proliferative and MMC treated fibroblasts were analyzed by RT-PCR for p16^ink4a^ and p21^Cip1^ as well as senescence phenotype using Senescence Associated β-galactosidase staining.

#### 4.1.3. Murine Ear Fibroblasts (MearFs)

Murine ear fibroblasts (MearFs) were obtained from C57BL/6J mice maintained in the INRCA “Specific Pathogen Free” (SPF) animal facility. The ear biopsies (2 mm radius) were obtained with a dedicated puncher, which minimized animal discomfort (Agnthos, Lidingö, Sweden), and served for identification purposes within the colony or for other projects. The recovered material (5 biopsies × 2 mm radius) was incubated in 40 mL 70% ethanol in a sterile 50 mL conical bottom tube for 5 min and then appropriately air-dried. Once dried, hair was removed, and ears were cut into smaller pieces using scissors. The cut tissues were then digested using a collagenase D-pronase solution (Sigma-Aldrich) and shaken at 200 rpm for 90 min at 37 °C. After 90 min incubation, the digested ear tissues were placed in a 70 μm cell strainer and forcefully grinded using a 10 mL syringe plunger for >5 min in a 10 cm cell culture dish with 10 mL complete medium. The cell suspension was then pipetted into a 15 mL conical bottom tube and spun for 7 min at ~580× *g* at 4 °C using a refrigerated cell centrifuge. Last, the supernatant was removed and 10 mL complete medium was added to the cell pellet in the 15 mL conical bottom tube and the cells were resuspended. The complete medium used was generated by adding to Roswell Park Memorial Institute medium (RPMI) 10% fetal calf serum (FCS), 50 µM 2-mercaptoethanol (Promega), 100 µM aspargine (Serva, Heidelberg, Germany), 2 mM glutamine (Gibco, Thermo Fisher Scientific, Waltham, MA, USA), 1% penicillin-streptomycin solution. Resuspended cells in 10 mL complete medium were seeded at a concentration of 7.5 × 10^5^ cells in T75 flasks (Corning Costar, Sigma Aldrich, St. Louis, MO, USA). Cells were maintained at 37 °C in a humidified 5% CO_2_ incubator. On the third day, the medium was replaced with 10 mL fresh complete medium containing 10 μL of amphotericin B to remove debris. When cells reached about 70% confluency, they were washed with PBS, detached with TrypLE (Thermo Fisher Scientific), and passaged.

For DNA damage-induced senescence, Mearf was treated with 75 nM Doxorubicin (DOX) or 75 nM mitomycin C (MMC) for 5–7 days and then incubated for 1 week in normal medium. For oxidative-stress-induced senescence, MearF were treated with 250 µM H_2_O_2_ for 2 h and then incubated for 1 week in normal medium.

For the radiation-induced senescence model, MearF cells were transported to the radiation facility of the ICS Maugeri (Pavia, Italy) in tissue culture flasks. The confluence of the culture prior to irradiation was almost 70%. Cells were subsequently exposed 10 Gy X-rays before being returned to standard culture conditions.

Senescent cells were then evaluated for the absence of proliferation and morphological alterations employing optical microscopy. SA-β-gal assay as well as RT-PCR for p16^ink4a^ and p21^Cip1^ were carried out as well. In the case of H_2_O_2_ treatment, we were unable to obtain a complete proliferation arrest, suggesting only a modest induction of senescence as revealed by morphological alterations and SA-β-gal assay.

For the experiments with senolytic compounds proliferating or senescent MearF were treated for 48 h with navitoclax (Cayman, KY, USA), fisetin (Cayman, KY, USA) or the combination of dasatinib (Cayman, KY, USA) and quercetin (Cayman, KY, USA) (DQ). These compounds were dissolved in DMSO (VWR, Milan, Italy) and subsequently diluted in cell culture medium to working concentrations of 10 μM (navitoclax, fisetin and quercetin) and 100 nM (dasatinib).

#### 4.1.4. Ex Vivo Cells from Mouse Ear Biopsies

For the analysis of cells taken directly from ear biopsies, we used 3 young male C57BL/6J mice (age 3 months) and 3 geriatric C57BL/6J mice (aged 30 months) that needed to be tagged twice in the same ear for identification purposes before being included in other experiments. One single biopsy of 2 mm from each mouse was used for IFC and another for SA-β-gal assay. For IFC analysis, the biopsy was rapidly washed with ethanol and immersed in the collagenase D-pronase solution for 90 min at 37 °C. The digested ear tissues were placed in a 70 μm cell strainer and forcefully grinded with the syringe plunger and 3 mL of PBS into a vial. The cell suspension was centrifuged for 7 min at ~580× *g* at 4 °C; then, the supernatant was carefully aspired, and the recovered cell pellet was suspended in 100 µL of PBS and directly analyzed. For senescence biomarkers assay, the ear biopsy was snap frozen in liquid nitrogen and stored for SA-β-gal assay as described in the section below dedicated to senescence biomarkers assays. 

#### 4.1.5. Ex Vivo Cardiac Pericytes

Cardiac pericytes were isolated from small fragments of human atria collected immediately after cardiac transplantation. Briefly, leftovers of donor atria and the whole explanted hearts were sent to the pathology lab by the cardiac surgeon operating at the Academic Hospital of Udine. Once in the lab, adipose tissue and epicardium were dissected with a scalpel and muscle tissue was minced, washed and digested with collagenase type 2 (Worthington, Lakewood, NJ, USA). Following digestion, isolated cells were washed, sieved to remove those with a diameter ≥40 µm and plated in a 100 mm-diameter fibronectin-coated plate (Sigma Aldrich) in a medium with 2% stem cell qualified FBS (Thermo Fisher Scientific), 10 ng/mL PDGF-BB, and 10 ng/mL EGF (both from Peprotech House, London, UK), as in [22]. Once reaching ≈ 70% confluence, cells were detached from the substrate and subcultured at about 4 × 10^3^ cells/mm^2^. Cells at the third/fourth passage were employed for all the assays. The study, conducted in accordance with the Declaration of Helsinki, was approved by the Ethics Committee of Udine (2 August 2011, ref. 47,831; 22 October 2013, ref. 58,635 and 1 August 2016, ref. 18,386).

### 4.2. Senescence Biomarkers Assays

#### 4.2.1. Senescence Associated β-Galactosidase Staining

Senescence-associated expression of β-Gal activity was detected using Senescence Detection Kit (BioVision Inc., Milpitas, CA, USA for HUVEC, HuDe and MSC; Sigma-Aldrich-QIA117 for MearF and ear biopsy tissues). For cell cultures: non-confluent cells cultured in 24-well plates or 12-well plates were fixed for 15 min at room temperature, then washed twice in PBS. Cells were incubated overnight at 37 °C with Staining Solution Mix (containing X-Gal). SA-β-gal was assessed by light microscopy. The percentage of positive cells was determined by counting at least 500 cells/well. For ear biopsy tissues: 10 μm-thick sections were prepared with a cryostat from snap-frozen tissues and mounted on SuperFrost Plus slides (VWR, Radnor, PA, USA). SA-β-gal staining was performed according to the manufacturer’s instructions of the Sigma-Aldrich-QIA117 staining kit (Sigma-Aldrich, St. Louis, MO, USA). Nuclei were counterstained with Nuclear Fast Red (NFR) (Sigma-Aldrich), and images (from 0.31 µM × 0.31 µM sections) were acquired using a Zeiss AxioCam HRc mounted on a Leitz Laborlux S light microscope. The percentage of senescent cells was determined as the mean of 3–4 images from each biopsy by counting the total and SA-β-gal-positive cells with the positive cell detection tool (setup parameters: Sigma 1.8, nuclear stain threshold 0.1, max background intensity 0.5, cell expansion 4 µM, cytoplasm threshold for SA-β-gal 0.2) available in the open source software for digital image analysis, QuPath v. 0.3.2 [53].

#### 4.2.2. p16ink4a and p21Cip1 qRT-PCR

Total RNA was extracted from cells using the RNeasy kit (Qiagen, Italy) according to the manufacturer’s instruction and quantified by NanoDrop spectrophotometer. cDNA synthesis from total RNA was performed using i-Script reverse transcriptase (Biorad, Hercules, CA, USA) according to the manufacturer’s guidelines. The resulting cDNA were subjected to real-time PCR assay to detect the expression levels of β-actin housekeeping gene as well as p16^ink4a^ and p21^Cip1^.

The primers used are reported in the supplementary data (Table S1). One µg of cDNA was amplified in a total volume of 20 µL containing iQ SYBR GREEN SUPERMIX (Bio-Rad, Hercules, CA, USA) on a BioRad iQ5 optical real-time PCR (Biorad, Hercules, CA, USA), employing primer concentrations of 150 nM for β-Actin and 200 nM for the other genes. For HuDe, 240 ng of total RNA from each sample were reverse-transcribed using iScript^TM^ cDNA Synthesis Kit (Bio-Rad, Hercules, CA, USA). qPCR for p21, p16 and GAPDH was performed with SYBR green dye (iTaqTMUniversal SYBR Green Supermix, Bio-Rad) on a CFX96 Maestro Connect (Bio-Rad). The primers for GAPDH and p21 and p16 are reported in the supplementary data (Table S1). Three biological and two technical replicates for each sample were performed.

Assays for each transcript were carried out as duplicates. Any inefficiencies in RNA input or reverse transcription were corrected by normalization to the housekeeping gene. Relative amounts of the target mRNAs were calculated based on the comparative CT method (ΔΔCt (Cycle Threshold)).

#### 4.2.3. KI-67 and γH2AX in Cardiac Pericytes

For immunofluorescence studies, cardiac pericytes were cultured on round glass coverslides (22 mm diameter) and fixed with 4% buffered paraformaldehyde, 20′, at room temperature. After fixation, cells were permeabilized with 0.1% TritonX 100 (Sigma-Aldrich) in PBS for 10′ at room temperature. Cells were then incubated with the anti-Ki67 antibody (Abcam, ab15580, 1:800 dilution) for 1 h at 37 °C. Following a 1 h incubation with an Alexa488 donkey anti-rabbit secondary antibody (Jackson ImmunoResearch, UK) at 37 °C, cells were incubated with the anti γH2AX antibody (Merck-Sigma, clone JBW30) for 1 h at 37 °C. Last, an Alexa555 donkey anti-mouse secondary antibody (Jackson Immuno Research) was incubated for 1 h at 37 °C to reveal the second staining. Slides were then mounted, employing the antifade mounting medium Vectashield containing DAPI (Vector laboratories, Newark, CA, USA).

### 4.3. FlowSight Analysis

The FlowSight system (Amnis, part of Merck Millipore, Seattle, WA, USA) is an advanced imaging flow cytometer, combining features of fluorescent microscopy and flow cytometry. The instrument is accompanied by a dedicated image analysis software (IDEAS), which allows advanced quantification of intensity, location, morphology, population statistics, and more, within tens of thousands of cells per sample. This allows feature finder tools for the identification of parameters that allow us to discriminate between two subpopulations based on the selection of few representative events. This instrument gives rise to novel applications that were difficult to achieve by either conventional flow cytometry or microscopy. All the IDEAS features used in this study (both the default features and those customized for our analysis) are described in Supplementary Table S2.

At least 50,000–100,000 cells in 100–200 µL were used for the acquisition and at least 7000 events in the high area vs. aspect ratio features of the brightfield image were acquired. The analysis was performed with the IDEAS 6.2 software. The instrument was operated with 405, 488, 642 and 785 nm lasers at 20, 60, 20 and 5.62 mW. To quantify the intensity of autofluorescence, we used the mean fluorescence intensity collected in Ch02 (band 480–560 nm). Width and height of the cells were estimated from the respective features implemented in IDEAS 6.2. Apoptotic cells and necrotic cells were identified and/or removed from the analysis based on Ch11 (band 640–745 nm) and Ch07 (435–505 nm) intensity after staining with Annexin APC (10 min prior acquisition) and DAPI (immediately before acquisition).

#### 4.3.1. Senescence Associated β-Galactosidase Flow Cytometry Assays

For the SA-β-gal assay, we measured the mean fluorescence intensity (with the 488 laser intensity set at 20 mW) corrected for autofluorescence in the high area vs. aspect ratio features of the brightfield image. Cells were incubated for 30 min in Bafilomycin A1 and then for 1 h at 37 °C, 5% CO_2_ with C_12_FDG as previously described [12]. They were then detached from the wells using TrypLE (Thermo Fisher Scientific), washed and resuspended in 100 μL PBS, and analyzed immediately with the Amnis Flowsight. For the Spider-β-Gal flow cytometry assay, cells were stained with the respective fluorogenic β-galactosidase detection kit (SPiDER-βGal, Dojindo) following the manufacturer procedures.

#### 4.3.2. Identification of Senescent Cells by Artificial Intelligence (AI) and Machine Learning (ML)

Cell populations were refined by applying two AI models in sequence using the Amnis AI software [54,55]. This process was devised to maximize analysis precision and avoid confounding elements depending on objects whose image was either clipped or consisted of aggregates of different sorts. Finally, cell senescence was quantified using a super-feature calculated by the Amnis ML module for IDEAS [54,56,57,58].

## Figures and Tables

**Table 1:** Observed changes in SI and % LAF in different models of senescence.

Model	Cell Type (Senescence Inducing Stress)	Expected Range of Senescent Cells Estimated by SA-β-Gal Assays	SI	% LAF
In vitro	MearF (DOX)	70–90%	+++	+++
In vitro	HuDe (MMC)	60–80%	+++	+++
In vitro	MearF (MMC)	60–80%	+++	++
In vitro	HUVEC (replicative stress)	70–90%	++	+++
In vitro	MearF (Irradiated, 8 days)	30–60%	++	++
In vitro	MSC (replicative stress)	60–80%	++	++
Ex vivo	Cardiac pericytes (heart failure)	25–40%	++	+
In vitro	MearF (H_2_O_2_)	10–40%	+	+
In vitro	MearF (replicative stress)	10–40%	+	+
In vivo	Ear skin biopsies (geriatric age)	1–3%	+	−
Any	Non senescent cells	0%	−	−

Mearf = Mouse ear Fibroblasts; HUVEC = human umbilical vein endothelial cells; MSC = bone marrow mesenchymal cells; HuDe = human dermal fibroblasts; DOX = Doxorubicin; MMC = Mitomycin C; SI = senescence index; LAF = % large autofluorescent cells; SA-β-gal = Senescence Associated beta-galactosidase. − = SI < 0.2; LAF < 15%. +++ = SI > 2.5; LAF > 50%. ++ = SI range 1–2.5; LAF range 30%–50%. + = SI range 0.3–1; LAF range 20–30%.

## Data Availability

The rough data of this study are available upon request to M.M. (m.malavolta@inrca.it).

## Supplementary Materials

The following supporting information can be downloaded at: https://www.mdpi.com/article/10.3390/cells11162506/s1, Figure S1: Morphology and senescence markers in proliferating and senescent cells; Figure S2: Gating strategy for the analysis of senescent and proliferating cells; Figure S3A: Quantitative estimation of problems in gating mixed population of senescent and non-senescent cells; Figure S3B: Cardiac pericytes in the high Area region, after cleaning of multiplets, are mostly positive to beta-galactosidase; Figure S3C: Irradiated and proliferating MearF in the high Area region, after cleaning of multiplets, are mostly positive to beta-galactosidase; Figure S4: Identification of parameters that can discriminate between proliferating and senescent cells using the feature finder tool of IDEAS; Figure S5: Autofluorescence and compensation of mouse ear fibroblasts (MearF) treated for 3 min with low (75 nM) and high (100 µM) concentration of Doxorubicin (DOX); Figure S6: Induction of senescence in mouse ear fibroblasts (MearF) by mitomycin (MMC) and ionizing radiation; Figure S7: Workflow to analyse imaging flow cytometry integrating AI and ML; Figure S8A: Schematic representation of the rationale underlying the first model of Amnis AI applied to cell images (HUVEC, in this example) for the removal from analysis of incomplete objects due to clipping; Figure S8B: Schematic representation of the of the rationale underlying the second model of Amnis AI applied in sequence to cell images (HUVEC, in this example) for the removal from analysis of unwanted aggregates of different sorts. Figure S9: Schematic representation of the of the rationale underlying the calculation of the IDEAS ML Classifier used to distinguish between senescent and non-senescent cells and quantification of cell senescence based on the ML classifier for representative examples; Figure S10: Identification of sub-populations based on population density of SI vs MLSC or MLSC2 scatter-plots; Table S1: Primer sequences used in RT-PCR; Table S2: List of parameters from the IDEAS software used in this study.
