# Supplementary material for: Simple Detection of Unstained Live Senescent Cells with Imaging Flow Cytometry
Source: Cells. 2022 Aug 12;11(16):2506. doi: 10.3390/cells11162506 (PMC9406876; doi:10.3390/cells11162506)
Supplement: Supplementary file 1 [file cells-11-02506-s001.zip › cells-1802566-supplementary.pdf]

Supplementary Data

Morphology and senescence markers in proliferating and senescent cells

Morphology and senescence markers in proliferating and senescent human bone marrow mesenchymal stem cells (MSC), mouse ear fibroblasts (MearF), human umbilical vein endothelial cells (HUVEC) and human dermal fibroblasts (HuDe) (**Supplementary Figure S1**).

On the left of each figure, are reported light microscope images of SA-β-GAL stained cells. On the right are reported RNA expressions of common senescence markers such as p16 and p21. Induction of senescence was performed by exposure to Doxorubicin (DOX) (1 week, 75 nM) in MearF, exposure to mitomycin C (MMC) (1 week, 75 nM) in HuDe and by replicative stress in HUVEC (passage 17, P17) and MSC (passage 15, P15). The histograms represent n = 3-6 independent replicates. MearF = mouse ear fibroblasts; HUVEC= human umbilical vein endothelial cells; MSC = human bone marrow mesenchymal stem cells. ** $p < 0.01$; *** $p < 0.001$ by paired student's t test.

Figure S1. Morphology and senescence markers in proliferating and senescent cells

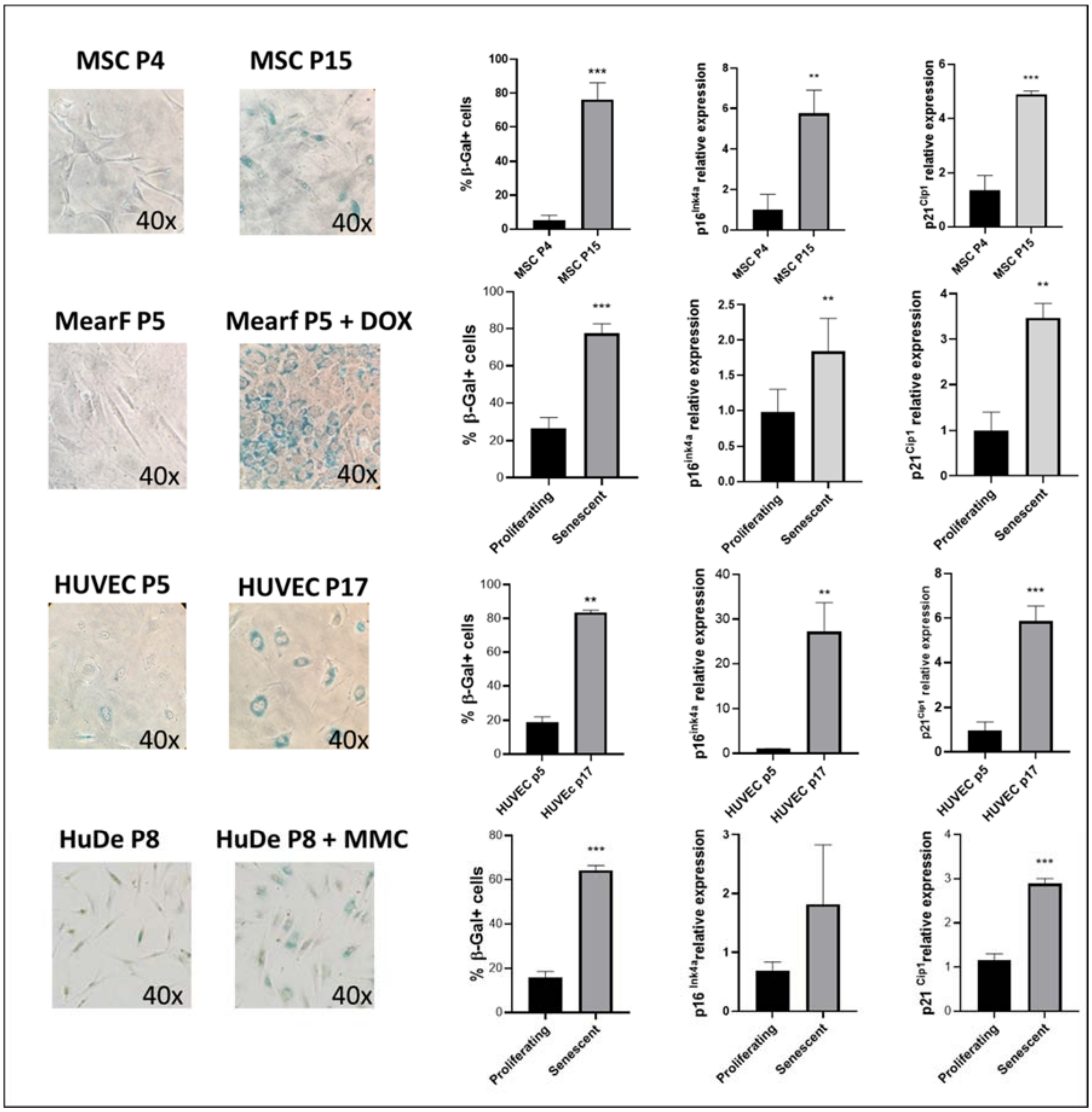

Gating strategy for the analysis of senescent and proliferating cells

Examples of our gating strategy applied to proliferating (P) and senescent (S) human mesenchymal stem cells (MSC), human dermal fibroblasts (HuDe), mouse ear fibroblasts (MearF). Senescent models included replicative senescence (RS), doxorubicin induced senescence (DOXS), as well as mitomycin C induced senescence (MMCS). a) P-MSC, b) RS-MSC, c) P-HuDe, d) MMCS-HuDe, e) P-MearF, f) DOXS-MearF, g) MMCS-MearF. The conventional “Single cells” gate used in imaging flow cytometry to select proliferating cells (red color) is replaced by a larger gate that includes also large cells and multiplets in the analysis (Single cells + large cells + multiplets gate, blue color). In order to clean the population from multiplets we applied a second gate (green gate) set in the high “Circularity” and high “Shape ratio”. Cell images below each gate represents the largest events detected in the respective gate. These images show that the application of the second gate removes almost completely the doubles and multiplets from the population that will be analyzed.

Figure S2. Gating strategy for the analysis of senescent and proliferating cells

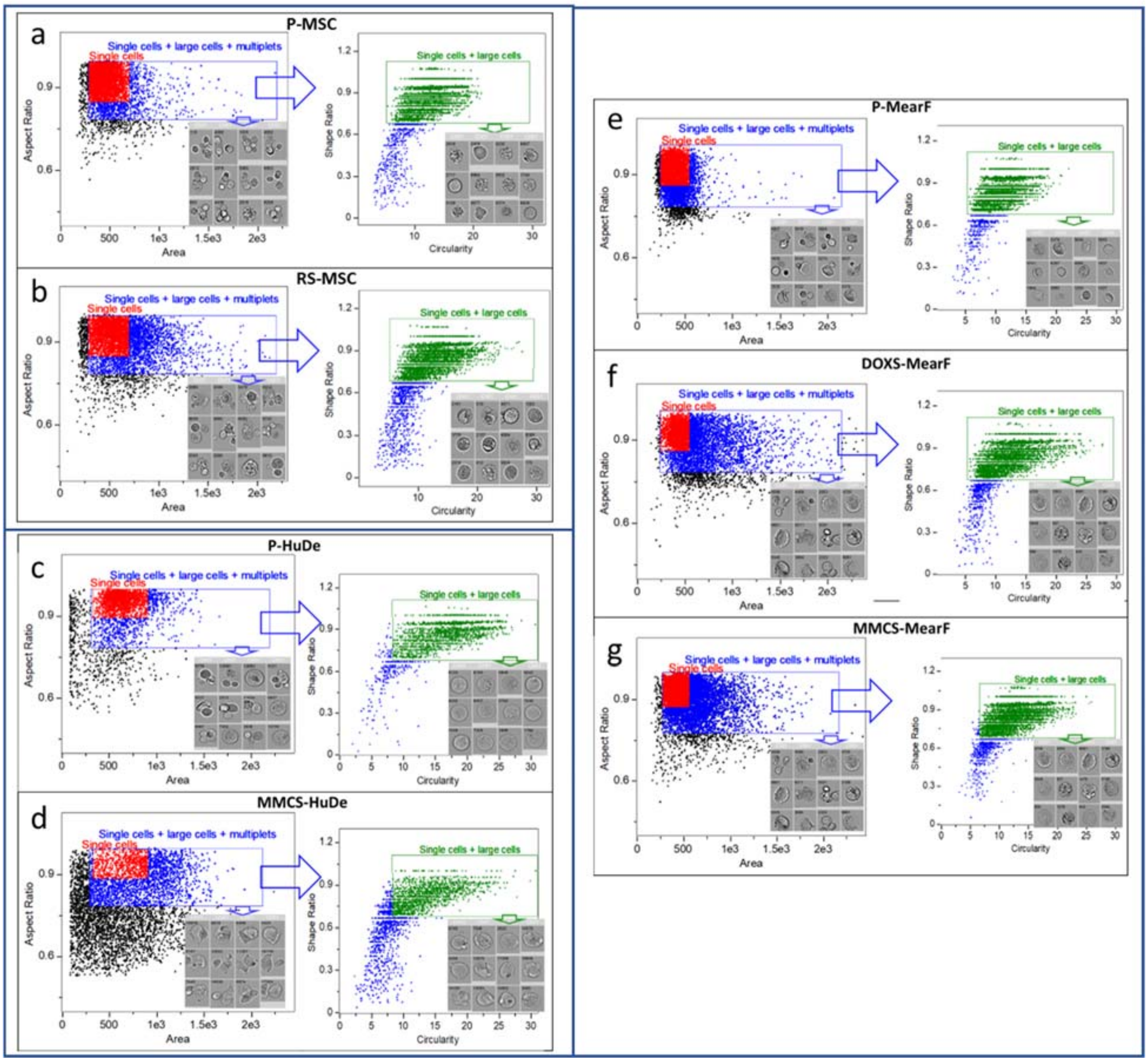

A quantitative example of the problem is provided from the analysis of a merged file from samples of proliferating (P) and replicative senescent (RS) HUVEC (**Supplementary Figure S3A**). Applying our gating strategy allows the inclusion in the analysis of all large cells with a negligible inclusion of multiplets and doublets (**Supplementary Figure S3A, Panel a**). b) A conventional Gate (Gate 1) drawn in the highest cell density region excludes quite completely multiplets from the analysis (0.03-0.1%) but a very high percentage of large cells (43.2% from the RS-HUVEC file) that are inside the dashed rectangle are also excluded from the analysis (**Supplementary Figure S3A, Panel b**). c) Extending the gate to the right (Gate 2) results in the loss of less large cells (10 % from the RS-HUVEC file) but also in the inclusion of an important amount of multiplets (2.7 % from the P-HUVEC file) (**Supplementary Figure S1A, Panel c**).

Using the "ex-vivo" pericyte model stained with Spider-βGAL we were able to provide evidence that cells in the high Area region are mostly positive to Spider-βGAL. In the example shown in **Supplementary Figure S3B** we have investigated the positivity to Spider-βGAL in the cells in the high area region of of cardiac pericytes (CPc) obtained from patients undergoing cardiac transplantation (E-CPc) or from healthy donors (D-CPc). In this example we found a 5.5% and 15.2% events in the high Area region, respectively in D-CPc and E-CPc. After cleaning of multiplets by gating in the high "Circularity" and high "Shape ratio" we observed that the cells in the high area region were 89.8 % and 99.6 % positive to Spider-βGAL, respectively in D-CPc and E-CPc. Notably, the whole population of D-CPc displayed only a 10% of cells positive to Spider-βGAL, while the whole E-CPc population displayed a 42.5% of positivity.

Very similar results were observed investigating the mean fluorescence intensity of Ch02 of the cells stained with Spider-βGAL. In another example, irradiated and proliferating MearF, the mean fluorescence intensity of Ch02 of the high area cells stained with Spider-βGAL was above 50000 (approx. 2-3 fold the mean fluorescence intensity of the whole population) in both proliferating and irradiated MearF (**Supplementary Figure S3C**).

Figure 3SA. Quantitative estimation of problems in gating mixed population of senescent and non-senescent cells

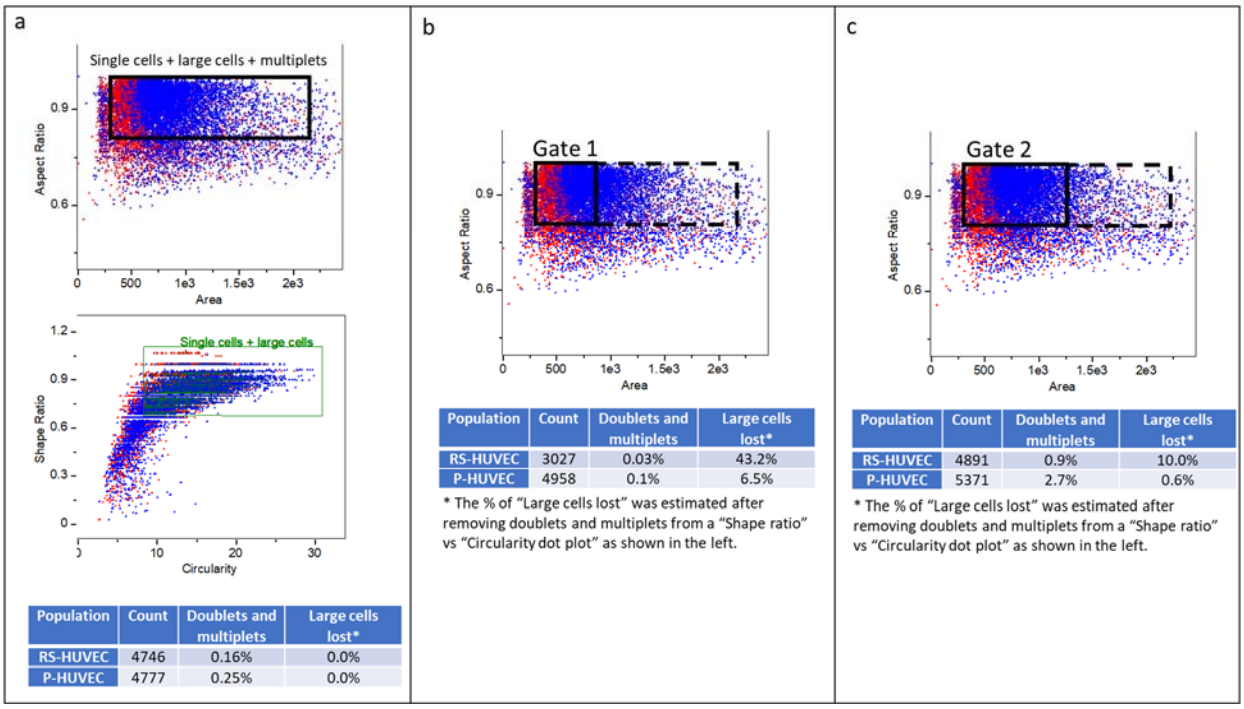

a

Population	Count	Doublets and multiplets	Large cells lost*
RS-HUVEC	4746	0.16%	0.0%
P-HUVEC	4777	0.25%	0.0%

b

Population	Count	Doublets and multiplets	Large cells lost*
RS-HUVEC	3027	0.03%	43.2%
P-HUVEC	4958	0.1%	6.5%

* The % of "Large cells lost" was estimated after removing doublets and multiplets from a "Shape ratio" vs "Circularity dot plot" as shown in the left.

c

Population	Count	Doublets and multiplets	Large cells lost*
RS-HUVEC	4891	0.9%	10.0%
P-HUVEC	5371	2.7%	0.6%

* The % of "Large cells lost" was estimated after removing doublets and multiplets from a "Shape ratio" vs "Circularity dot plot" as shown in the left.

Figure S3B. Cardiac pericytes in the high Area region, after cleaning of multiplets, are mostly positive to beta-galactosidase.

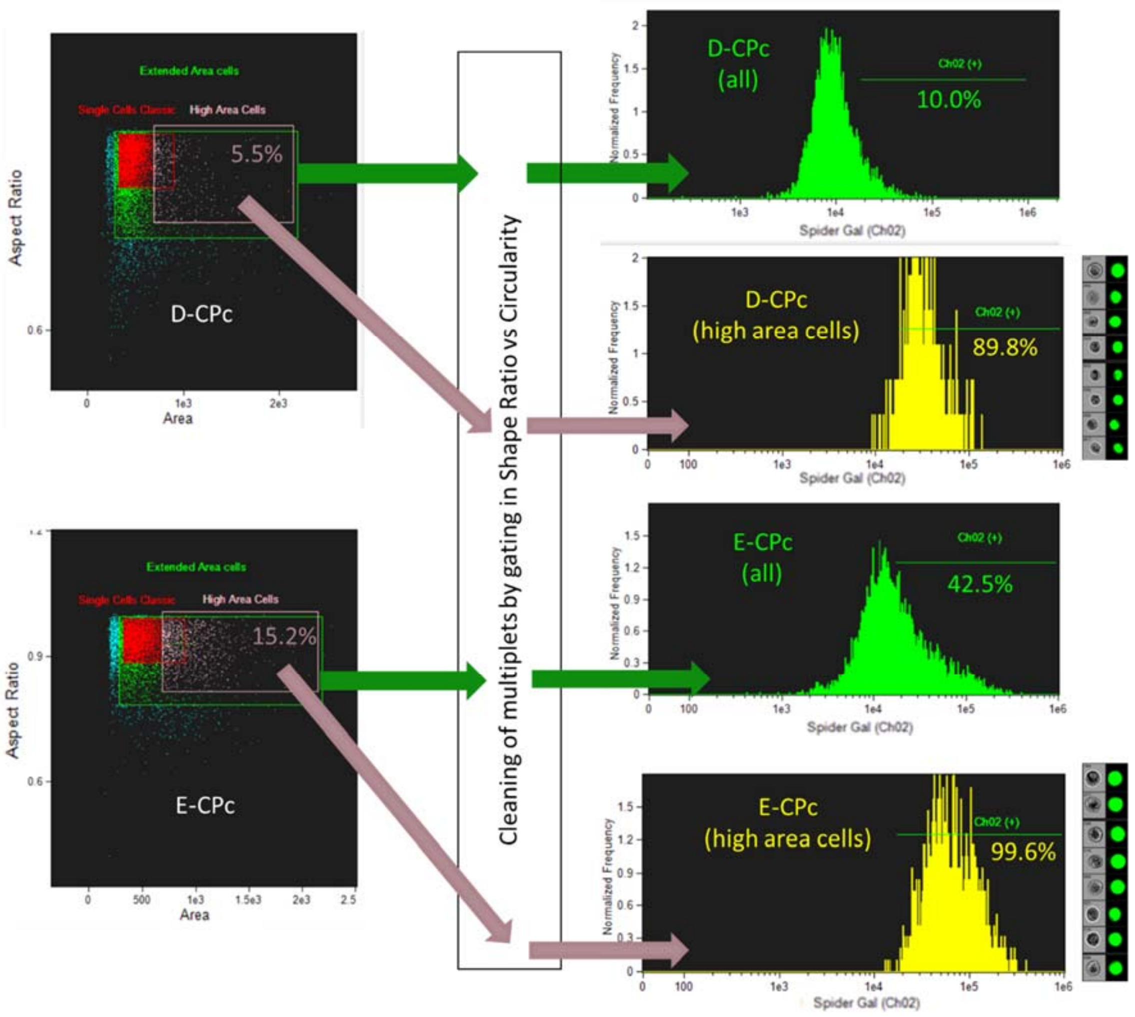

Figure S3C. Irradiated and proliferating MearF in the high Area region, after cleaning of multiplets, are mostly positive to beta-galactosidase.

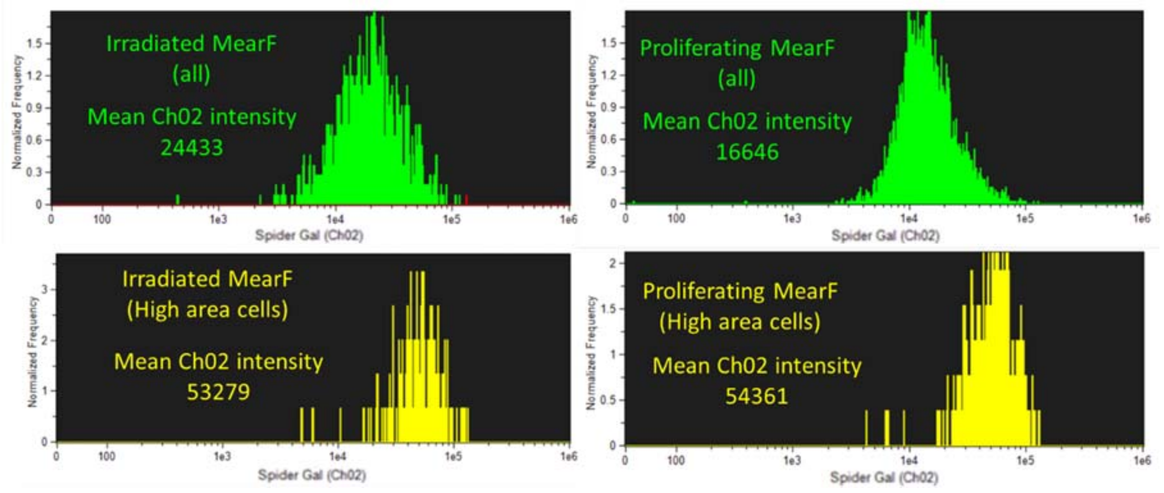

Identification of parameter to discriminate senescent vs non senescent cells using the feature finder tools

The Feature finder tool (available in all versions of the IDEAS software) was applied to merged .cif files of the "live cells" populations of senescent (green) and non-senescent (red) samples. "True senescent" and "true non-senescent cells" were picked in not overlapped regions from the extreme left and right of the "Area vs Aspect Ratio" (**Supplementary Figure S4**). Representative examples of the top features ranked by the Feature finder tool Feature for each model are reported below. The top 3 features (all with scores above 1.5) are underlined in red (**Supplementary Figure S4**).

Figure S4. Identification of parameters that can discriminate between proliferating and senescent cells using the feature finder tool of IDEAS

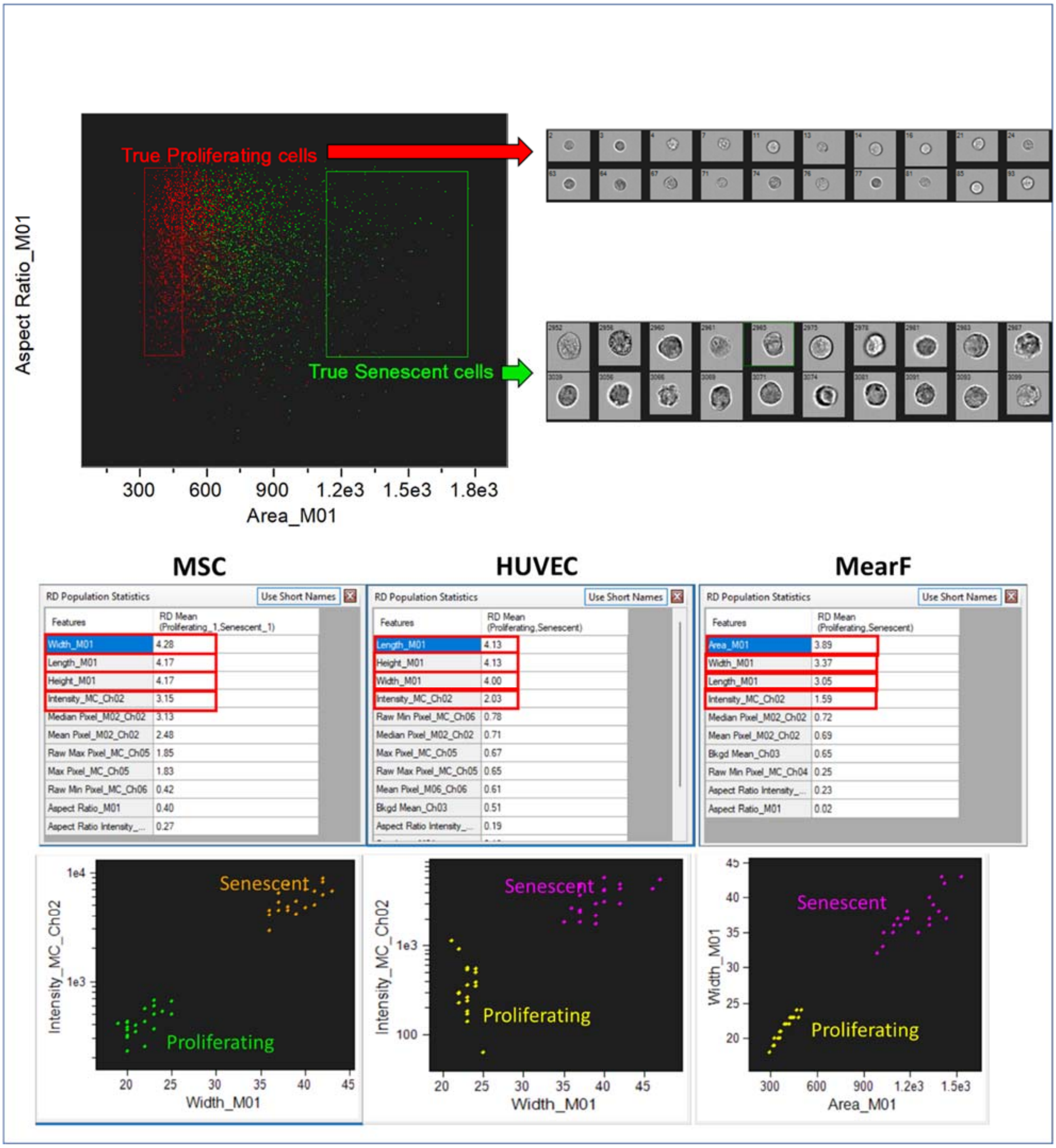

Compensation of Doxorubicin (DOX) intrinsic autofluorescence

Estimation and compensation of Doxorubicin (DOX) intrinsic autofluorescence (**Supplementary Figure S5**) in MearF. a) Representative histogram of the intensity of AF collected in Ch02 (top) and Ch04 (bottom) in untreated MearF. b) Representative histogram of the intensity of AF collected in Ch02 (top) and Ch04 (bottom) in MearF treated for 3 min with 75 nM DOX. c) Representative histogram of the intensity of AF collected in Ch02 (top) and Ch04 (bottom) in MearF treated for 3 min with 100 µM DOX. d) Representative histogram of the intensity of AF collected in Ch02 (top) after compensating for the "spillover" of Ch04 in Ch02.

Figure S5. Autofluorescence and compensation of mouse ear fibroblasts (MearF) treated for 3 min with low (75 nM) and high (100 µM) concentration of Doxorubicin (DOX).

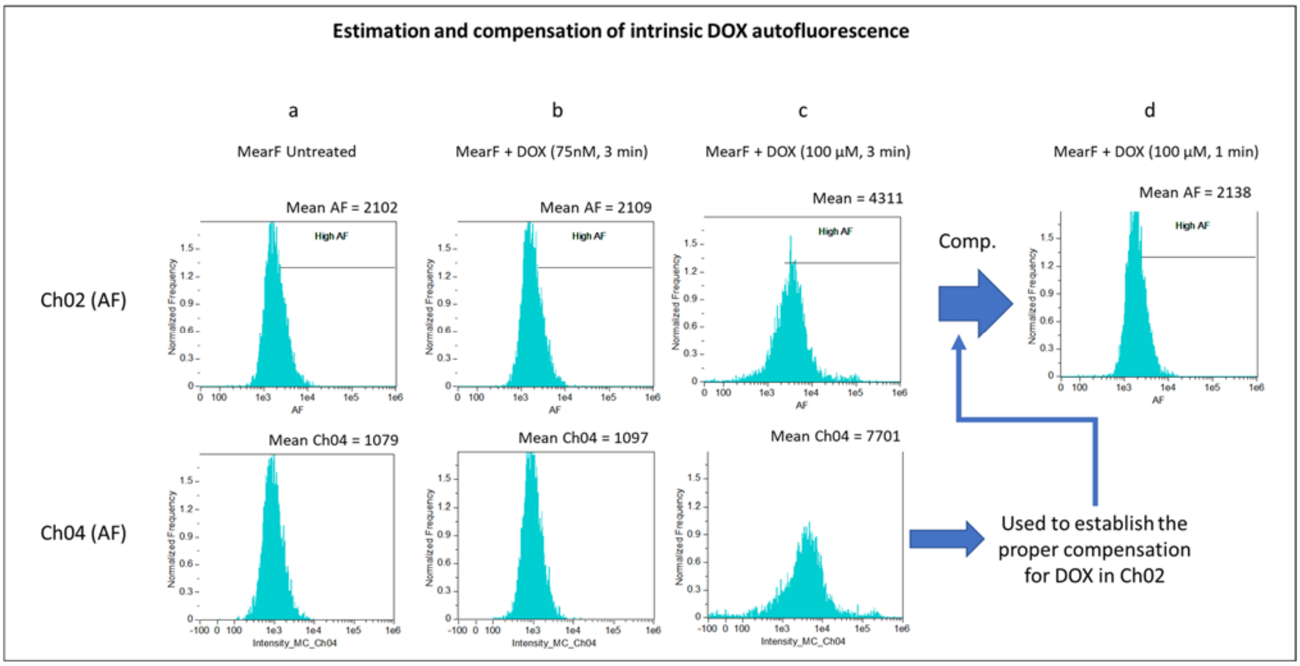

Induction of senescence in MearF by mitomycin (MMC) and ionizing radiation

Induction of senescence in MearF by mitomycin (MMC) (75 nM for 1 week) (**Supplementary Figure S6a–d**): a) Representative histogram showing autofluorescence (AF), mean diameter (D), large autofluorescent cells (LAF) and S-index (SI) in senescent MearF. All these parameters were similar to those observed for DOX induced senesce as reported in **Figure S2** of the manuscript. b) Representative histogram of senescent MearF treated for 3 min with 100 µM MMC showing that this compound do not display autofluorescence properties per se (paradoxically, the overall AF signal slightly decreases likely due to cell death or to other unknown effects related to this massive addition). c) staining of SA-β-Gal in MearF treated with MMC for 1 week showing an increased staining compared to the controls of **Supplementary Figure S1**). d) LAF and SI measured in four replicate samples of MearF induced to senescence by MMC vs control MearF ($p < 0.001$). The mean levels of SI and LAF after treatment with MMC are slightly below but comparable to the values obtained after treatment with DOX. Induction of senescence in MearF by ionizing radiation (10 Gy) (**Supplementary Figure S6e–g**): e) Representative histogram showing autofluorescence (AF), mean diameter (D), large autofluorescent cells (LAF) and S-index (SI) in irradiated MearF (10 Gy, 8 days post irradiation). All these parameters were significantly higher compared to proliferating cells, but lower than those observed for DOX and MMC induced senesce as reported in Fig. 2 and Suppl. Fig. 6A. b) Spider-βGal staining of irradiated and proliferating MearF showing a partial induction of senescence after ionizing radiation (around 40 %) ($p < 0.05$ for mean fluorescence intensity and $p < 0.01$ for % of positive cells). c) LAF and SI measured in three replicate samples of irradiated (days 3 and days 8 post irradiation) and proliferating MearF. The mean levels of SI and LAF are significantly higher in irradiated vs proliferating MearF both 3 days ($p < 0.01$) and 8 days ($p < 0.001$) post irradiation.

Figure S6. Induction of senescence in mouse ear fibroblasts (MearF) by mitomycin (MMC) and ionizing radiation

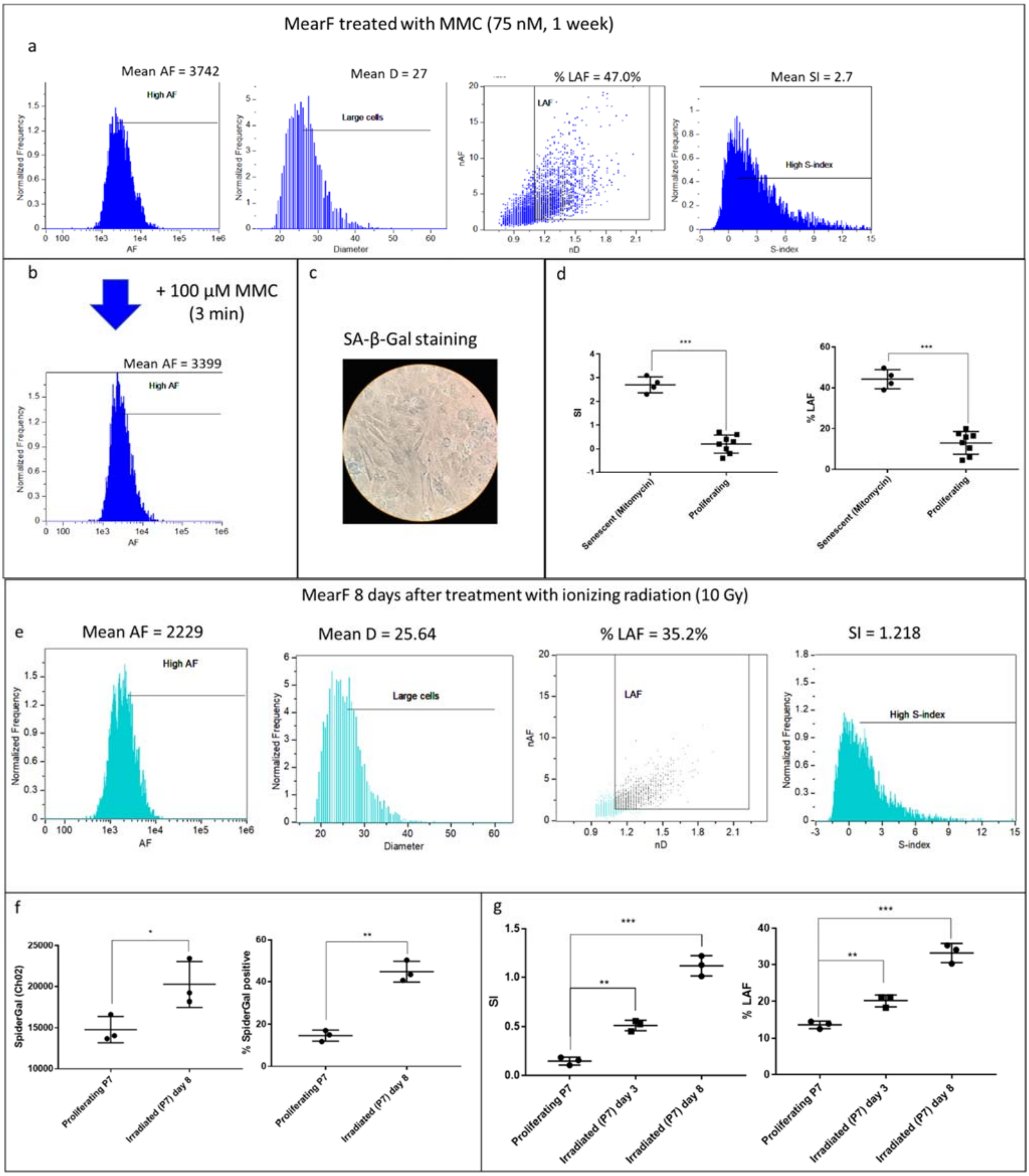

Quantification of senescent cells estimated by artificial intelligence (AI) and machine learning (ML)

A schematic representation of the workflow followed to analyse imaging flow cytometry data integrating AI and ML (**Supplementary Figure S7**).

A schematic representation of the rationale underlying the first model of Amnis AI applied to cell images (HUVEC, in this example) for the removal from analysis of incomplete objects due to clipping is shown in **Supplementary Figure S8A**. On the left (Exported Live Cells, see materials and methods), an example of variegated cell images included in the initial datafile. These include properly acquired whole objects (# 3769 and 3423) and unusable partial ones (# 1752 and 5631: Clipped objects). The classification of cells in the datafile with Amnis AI allowed to correctly separate objects in two subpopulations: Usable and Clipped objects. After double checking of the results, the first, have been kept for further analysis while the second have been discarded.

A schematic representation of the of the rationale underlying the second model of Amnis AI applied in sequence to cell images (HUVEC, in this example) for the removal from analysis of unwanted aggregates of different sorts is shown in **Supplementary Figure S8B**. On the left, an example of non-clipped cell images obtained after their classification with Amnis AI Model 1 (Exported Cells From Model 1, see Figure SA from supplementary information). These include single cells (# 1294, 81 and 393) and unusable aggregates (#904, 1294, 533 and 2246: Aggregate objects). The classification of cells in the datafile with Amnis AI allowed to correctly separate objects in two subpopulations: Usable and Aggregate objects. After double checking of the results, the first, have been kept for further analysis while the second have been discarded. The table at the bottom indicates that the average precision of the model is above 95%.

A Schematic representation of the of the rationale underlying the calculation of the IDEAS ML Classifier used to distinguish between senescent and non-senescent cells is shown in **Supplementary Figire S9**.

Supplementary Figure S9, Panel A) After Amnis AI classification, new datafiles were generated by merging together usable cells from non-senescent and senescence-induced samples. The resulting datafile (Doxorubicin treated mouse fibroblasts in this example) was analysed by applying the analysis template used to measure the S-index of single cell (see materials and methods). The S-index was then used to define two truth populations: Non senescent cells, coming from the non-senescent part of the merged datafile and characterized by S-index values ≤ 0.8 and Senescent cells, (green marker, left histogram) consisting of objects from the senescent part of the merged datafile and displaying S-index values ≥ 1.2 (red marker, right histogram). Based on these, a ML Senescence Classifier (or super feature) was calculated by the Amnis IDEAS Machine Learning module in order to obtain the maximal statistical separation between the two truth populations as shown in the overlayed histogram at the bottom (non-senescent: green vs senescent: red). The table on the right indicates the top five single features incorporated in the ML Senescence Classifier for mouse fibroblasts, based on their relative statistical power of separation.

Supplementary Figure S9, Panel B) Quantification of cell senescence based on the ML Senescence Classifier for representative samples of non-senescent (left) and senescence-induced (right) cells. Each row of paired histograms corresponds to a specific cell type. These are respectively: Mouse ear fibroblasts (MearF), human umbilical vein endothelial cells (HUVEC), human bone marrow mesenchymal stem cells (MSC), Human Pericytes (E-CPc: from patients undergoing cardiac transplantation; D-CPc from healthy donors) and Human dermal fibroblasts (HuDe).

Based on the extreme events from the population density observed in the SI vs MLSC scatter plots we also calculated a second classifier (MLSC2) and plotted SI vs MLSCs. The scatter plots of Si vs MLSCs provided a better separation between senescent and non-senescent cells and uncovered the presence of various sub-populations in replicative senescent models (**Supplementary Figure S10**)

Figure S7. Workflow to analyse imaging flow cytometry integrating AI and ML.

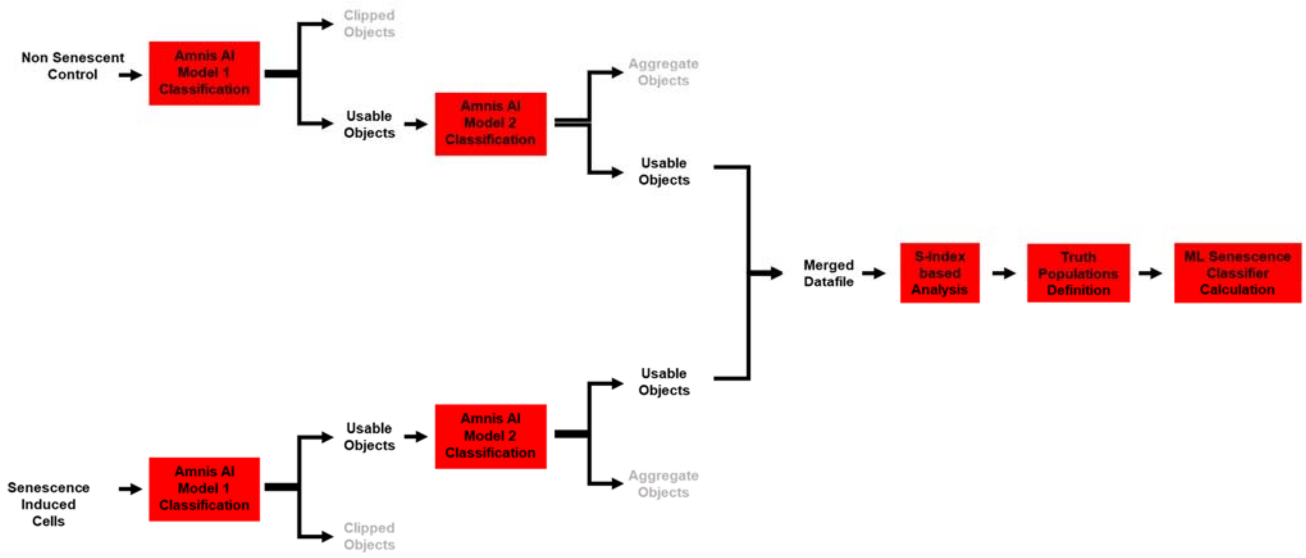

Figure S8A. Schematic representation of the rationale underlying the first model of Amnis AI applied to cell images (HUVEC, in this example) for the removal from analysis of incomplete objects due to clipping.

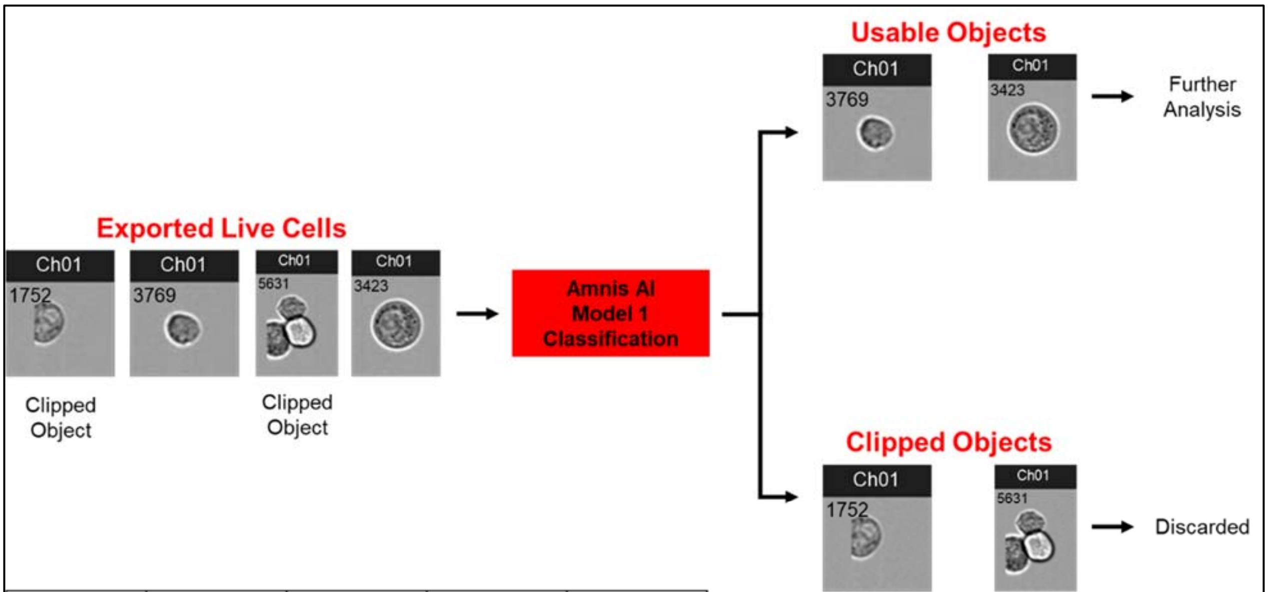

Class	Precision (%)	Recall (%)	F1 (%)	Support
Clipped	75.57	85.90	80.41	922
Usable	99.02	98.09	98.55	13410
Weighted avg	97.51	97.31	97.39	14332

Figure S8B. Schematic representation of the of the rationale underlying the second model of Amnis AI applied in sequence to cell images (HUVEC, in this example) for the removal from analysis of unwanted aggregates of different sorts.

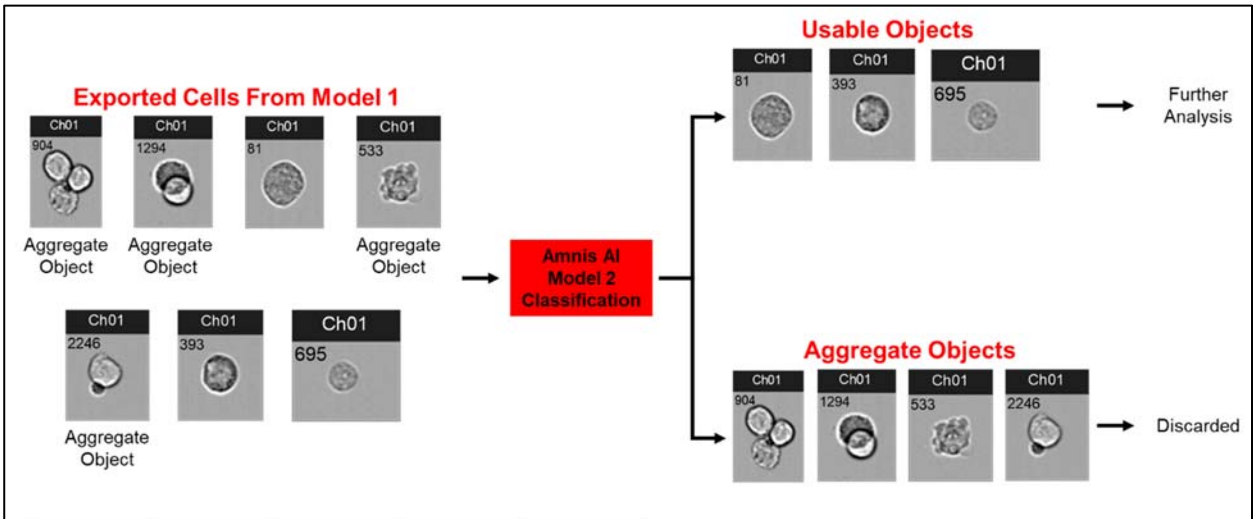

Class	Precision (%)	Recall (%)	F1 (%)	Support
Aggregate	73.87	64.81	69.04	807
Usable	97.48	98.35	97.91	11193
Weighted avg	95.90	96.09	95.97	12000

Figure S9. Schematic representation of the of the rationale underlying the calculation of the IDEAS ML Classifier used to distinguish between senescent and non-senescent cells and quantification of cell senescence based on the ML classifier for representative examples

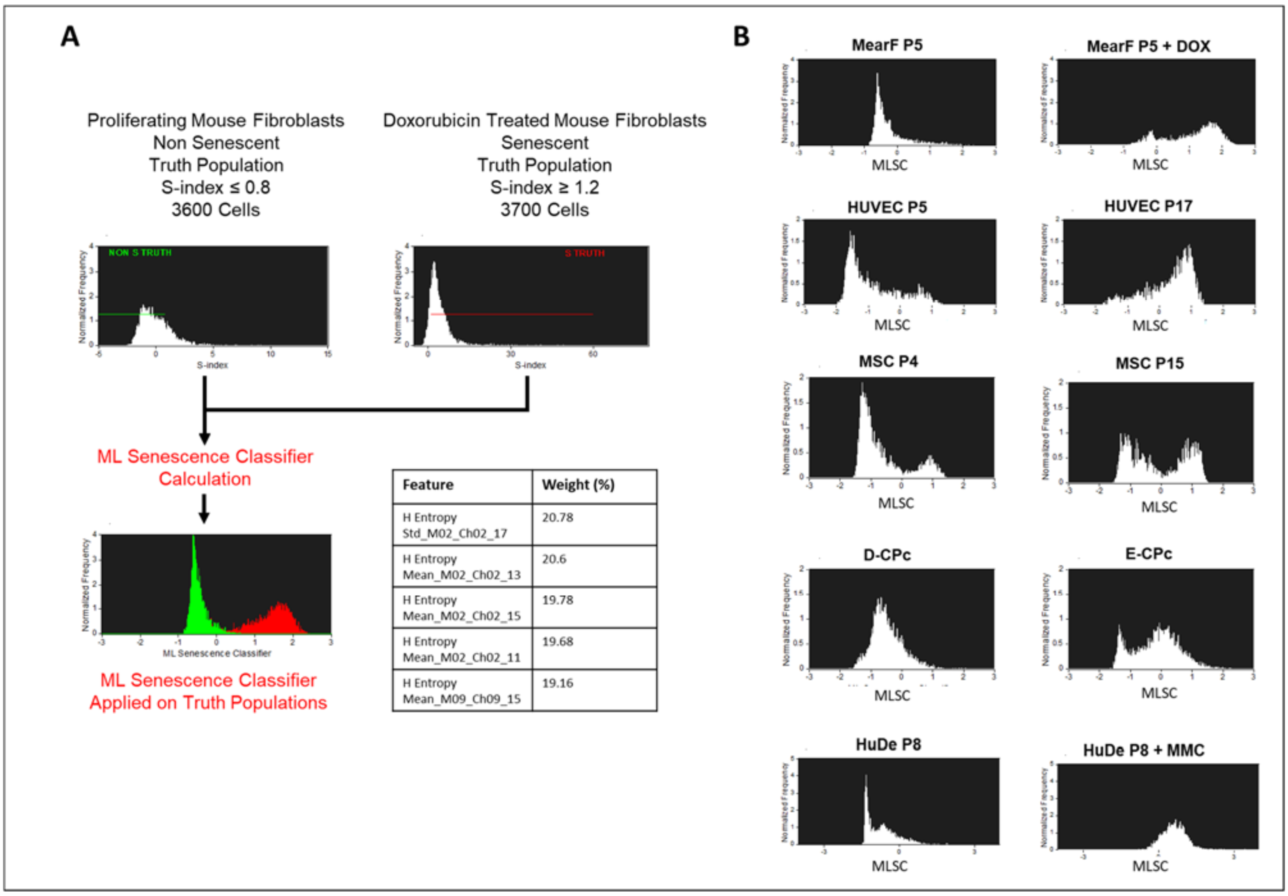

Feature	Weight (%)
H Entropy Std_M02_Ch02_17	20.78
H Entropy Mean_M02_Ch02_13	20.6
H Entropy Mean_M02_Ch02_15	19.78
H Entropy Mean_M02_Ch02_11	19.68
H Entropy Mean_M09_Ch09_15	19.16

Figure S10. Identification of sub-populations based on population density of SI vs MLSC or MLSC2 scatter-plots

A

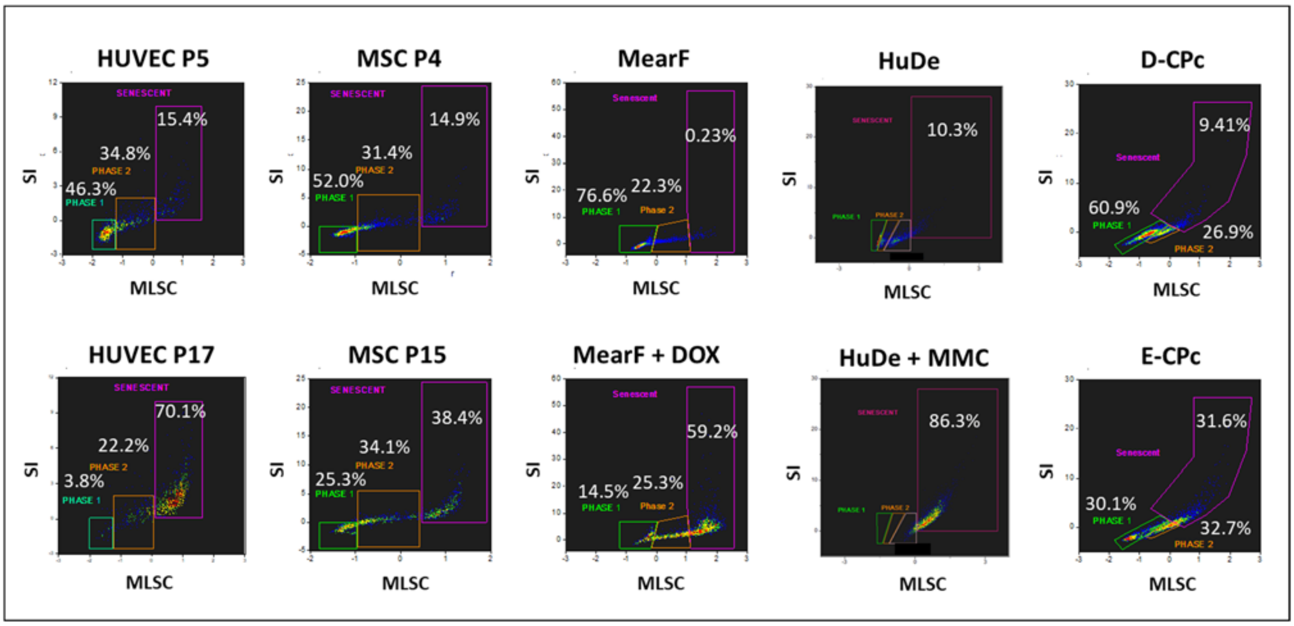

B

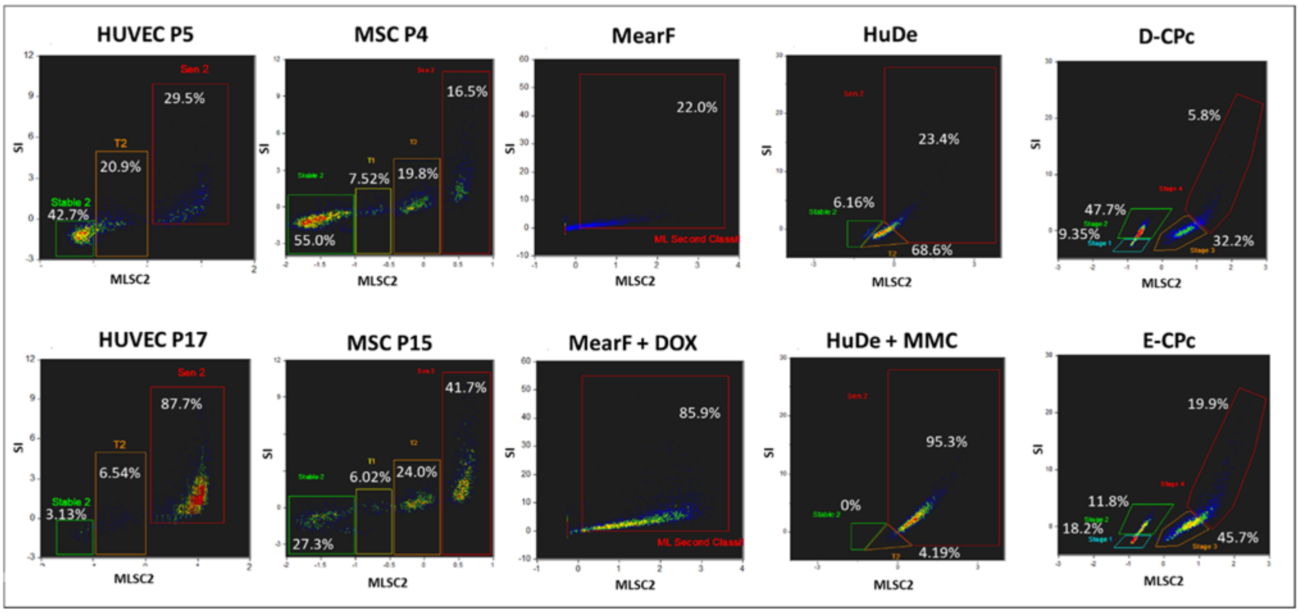

Table S1. Primer sequences used in RT-PCR

	Forward primer	Reverse primer
**mouse p16**	5′-CGT ACC CCG ATT CAG GT-3′	5′-TTG AGC AGAAGA GCTGC TACGT-3′
**mouse p21**	5′-GCT GTCTTGCAC TCTGGTGT-3′	5′-TCTGCGCTTGGAGTGATAGA-3′
**mouse β-actin**	5′-TTCGTTGCCGGTCCACAC-3′	5′-ACCAGCGCAGCGATATCG-3′
**Human p16 (HUVEC and MSC)**	5'- GAGCAGCATGGAGCCTTC-3'	5'-CATCATCATGACCTGGATCG-3'
**Human p21 (HUVEC and MSC)**	5′-GGAAGACCATGTGGACCTGT-3′	5′-GGCGTTTGGAGTGGTAGAAA-3′
**Human β-actin (HUVEC and MSC)**	5′-GGATAGCACAGCCTGGATAG-3′	5′-GCGAGAAGATGACCCAGATC-3′
**Human p16 (HuDe)**	5′- CATAGATGCCGCGGAAGGT-3′	5′-CTAAGTTTCCCGAGGTTTCTCAGA-3′
**Human p21 (HuDe)**	5′-CCATCCCTCCCCAGTTCATT-3′	5′-AAGACAACTACTCCCAGCCC-3′
**Human GAPDH (HuDe)**	5′-TCCACTGGCGTCTTCACC-3′	5′-GGCAGAGATGATGACCCTTTT-3′

Table S2. List of parameters from the IDEAS software used in this study.

Default features	Description
Area	The number of microns squared in a mask is equal to the Area. To obtain that, the number of pixels is converted to µm2. For instance, at 40X magnification 1 pixel = 0.25 µm2. As an example, a cell with a mask that includes 2000 pixels is therefore equal to 500 µm2.
Aspect Ratio	Aspect Ratio is the Minor Axis divided by the Major Axis of the image and describes how round or oblong an object is.
Circularity	This feature measures the degree of the mask's deviation from a circle. Its measurement is based on the average distance of the object boundary from its center divided by the variation of this distance. Thus, the closer the object to a circle, the smaller the variation and therefore the feature value will be high. Vice versa, the more the shape deviates from a circle, the higher the variation and therefore the Circularity value will be low.
Height and Width	Using the bounding rectangle, Height is the number of microns of the longer side and Width the shorter side<br>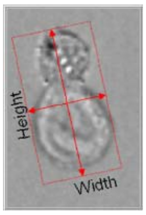
Intensity	The Intensity feature is the sum of the background subtracted pixel values within the masked area of the image.
Thickness Min	Thickness Min measures the smallest width of an object. This feature is based on an input mask and therefore sensitive to the variation of the input mask shape. Selecting an input mask that can accurately capture the object shape is important.

Length	Length measures the longest part of an object. Unlike the Major Axis feature, Length can measure the object's length even if it folds to form a cashew, banana, or doughnut shape, where in many of these cases the major or minor axis features would not be able to differentiate these with true circular shaped objects with no hole.
Shape ratio	The shape ratio is computed based on length and thickness min features. The Shape Ratio feature is based on an input mask and is sensitive to the variation of the input mask shape. Selecting an input mask that can accurately capture the object shape is important. 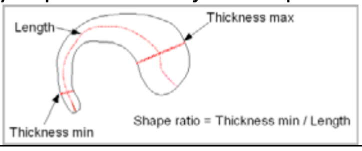
**Customized feature**	**Description**
D (Diameter)	(Width + Height)/2
AF (Autofluorescence)	The intensity of of the cell in channel 2 without any staining
nAF	The normalized AF: the ratio of the AF of the sample to the AF of normal proliferating cells
nD	The normalized D: the ration of the D of the sample to the D of normal proliferating cells
S-index (SI)	A senescence index computed as follows: (SI = ((nAF-1) + 5*(nD-1))/2)

* Default features are those available in all versions of IDEAS. Customized features were computed from default features using the calculation tool available in IDEAS.
